# CRISPR-Cas9 Toolkit for Genome Editing in an Autotrophic CO_2_-Fixing Methanogenic Archaeon

**DOI:** 10.1128/spectrum.01165-22

**Published:** 2022-06-29

**Authors:** Jie Li, Liuyang Zhang, Qing Xu, Wenting Zhang, Zhihua Li, Lei Chen, Xiuzhu Dong

**Affiliations:** a State Key Laboratory of Microbial Resources, Institute of Microbiology, Chinese Academy of Sciences, Beijing, China; b Center for Life Sciences, School of Life Sciences, Yunnan University, Kunming, China; c Laboratory of Synthetic Microbiology, School of Chemical Engineering & Technology, Tianjin University, Tianjin, China; d University of Chinese Academy of Sciences, Beijing, China; South China Sea Institute of Oceanology

**Keywords:** CRISPR-Cas9, genome editing, archaea, CO_2_ fixing, methanogenic archaeon, genetics, *Methanococcus maripaludis*

## Abstract

The CRISPR-Cas9 system is a robust genome editing tool that is widely applied in eukaryotes and bacteria. However, use of this technique has only been developed for one species of *Archaea*, a domain of life ranking in parallel with *Eukarya* and *Bacteria*. In this study, we applied the CRISPR-Cas9 genome editing technique to Methanococcus maripaludis, an autotrophic and hydrogenotrophic methanogenic archaeon with a remarkably polyploid genome comprising up to ~55 chromosomal copies per cell. An editing plasmid was designed that encodes small guide RNA (sgRNA), Cas9 protein and an ~1-kb repair template (donor). Highly efficient (75% to 100%) and precise genome editing was achieved following one-step transformation. Significantly, the Cas9-based system efficiently deleted one or two genes and a large DNA fragment (~9 kb) and even synchronously deleted 13 genes located at three loci in all chromosomal copies of *M. maripaludis*. Moreover, precise *in situ* genome modifications, such as gene tagging and multiple- and even single-nucleotide mutagenesis, were also introduced with high efficiency. Further, as a proof of concept, precise mutagenesis at the nucleotide level allowed the engineering of both transcriptional and translational activities. Mutations were introduced into an archaeal promoter BRE (transcription factor B [TFB] recognition element), a terminator U-tract region, and a gene coding region. Stop codon introduction into a gene through single-nucleotide substitution shut down its expression, providing an alternative strategy for gene inactivation. In conclusion, the robust CRISPR-Cas9 genetic toolkit developed in this investigation greatly facilitates the application of *M. maripaludis* as a model system in the study of archaeal biology and biotechnology development, particularly CO_2_-based biotechnologies.

**IMPORTANCE** Archaea are prokaryotes with intriguing biological characteristics. They possess bacterial cell structures but eukaryotic homologous information processing machinery and eukaryotic featured proteins. Archaea also display excellent adaptability to extreme environments and play pivotal roles in ecological processes, thus exhibiting valuable biotechnological potential. However, the in-depth understanding and practical application of archaea are much lagging, because only a minority of pure cultures are available, and even worse, very few can be genetically manipulated. This work developed CRISPR-Cas9-based genome editing technology in Methanococcus maripaludis, a CO_2_-fixing methanogenic archaeon. The CRISPR-Cas9 approach developed in this study provides an elegant and efficient genome editing toolkit that can be applied in the knockout of single or multiple genes, *in situ* gene tagging, multiple- or single-nucleotide mutagenesis, and inactivation of gene expression by introduction of stop codons. The successful development of the CRISPR-Cas9 toolkit will facilitate the application of *M. maripaludis* in archaeal biology research and biotechnology development, particularly CO_2_-derived biotechnologies.

## INTRODUCTION

CRISPR-Cas (clustered regularly interspaced palindromic repeats and the associated *cas* genes) systems naturally function as adaptive immunity mechanisms in ~90% of archaeal and ~40% of bacterial species. They confer upon their hosts acquired immunity to phages and foreign DNA elements (1–3). In recent years, CRISPR-Cas systems have been developed as the best-known key components of a new generation of genome engineering tools (1–4). Among various types of CRISPR-Cas systems, the Cas9-based type II system from Streptococcus pyogenes is particularly well characterized and has been extensively developed as a noteworthily effective genome editing tool in various organisms, especially in eukaryotes (1, 2, 4) and a wide range of bacterial species (4–7). However, to date, the implementation of the Cas9 genome editing tool has only been successfully implemented in one archaeon, Methanosarcina acetivorans (8). This hinders the mechanistic understanding of the enormous diversity of archaeal biology and the translation of their potential in biotechnology.

*Archaea* represent the third form of cellular life, ranking in parallel with the *Bacteria* and *Eukarya* domains. Since proposed by Woese in 1977 (9, 10), archaea have been found to be ubiquitously distributed in every corner of Earth, particularly in various extreme environments. They are therefore believed to define the boundary of the biosphere and play pivotal roles in the biogeochemical recycling of carbon, nitrogen, sulfur, and other elements on Earth (11–15). The dramatic and more newly identified metabolic capabilities and potentials of archaea have further suggested the value of biotechnological applications of this group of organisms (16–19). Moreover, the simplified machineries of replication, transcription, and translation employed by archaea being homologous to those of eukaryotes (20, 21), and the archaeal ancestor of eukaryotes being illuminated by the recently identified Asgard archaea (22–24), further indicate the significant values of archaea in disclosing the evolutionary trajectory of life, particularly in regard to the mechanisms of genetic information processing. Therefore, archaea possess pivotal scientific value in the study of genetics, evolution, and metabolism as well as vast potential for a wide range of biotechnological applications (15, 16). However, compared to the case with bacteria and eukaryotes, scientific knowledge about archaeal biology and biotechnology is much lagging. This could be largely attributed to the facts that most archaeal species are recalcitrant to laboratory cultivation and, even worse, only a tiny proportion of the cultured species possess genetic systems that facilitate the study of their biology (20, 21).

Methanogenic archaea are a featured archaeal group characterized by the production of methane, which is the only energy conservation pathway of these archaea. They are also the only group of organisms producing ample methane and contribute 70% of the 500 to 600 Tg of annual global methane released into the atmosphere (25, 26). Uniquely, methanogens utilize simple substrates such as H_2_ and CO_2_, formate, acetate, and methylated compounds to generate methane and consequently implement the final step of biomass degradation and convert the metabolic wastes of organisms, including plants, algae, and cyanobacteria to methane in natural environments. Significantly, due to the utilization of simple and inexpensive metabolic substrates and the production of methane, a renewable and clean biofuel, the potential applications of methanogens have attracted great attention, particularly in development of CO_2_-based biotechnology (25, 27, 28). Therefore, conventional genetic tools have been separately developed in two distantly related genera: the mesophilic *Methanococcus* and *Methanosarcina*. These tools include DNA transformation, replicative and suicide vectors, positive and negative selection markers, transposon insertion, and reporter genes (25, 29, 30). Recently, further advances have been achieved, like the p5L-R-based markerless knockout system developed in *Methanococcus* (31) and a Cas9-based genome editing tool for *Methanosarcina* (8, 32), which facilitate more efficient gene knockout, tagging, and knockdown.

Methanococcus maripaludis is a rapidly growing, fully sequenced, and genetically tractable archaeal representative species, especially among the hydrogenotrophic methanogens (28, 29). Its capability of converting CO_2_ and H_2_ into CH_4_ and its fast autotrophic growth on CO_2_ have made *M. maripaludis* an attractive archaeal chassis in inexpensively producing high-value biochemical products, such as hydrogen, methanol, and geraniol (16, 27), indicating the high potential of *M. maripaludis* as a superior CO_2_-fixing cell factory for fundamental and experimental biotechnology research. This species is also regarded as a model archaeon in archaeal biology research, such as in transcription and posttranscription regulation (33–35), motility characteristics (36, 37), featured CO_2_ and N_2_ fixation, and methanogenic metabolism (38–45). By taking advantage of its genetic system, we previously found the long-sought transcription termination factor aCPSF1 and its mediated archaeal termination mechanism, which represents a bacterium-distinct and simplified archetypal mode of eukaryotic RNA polymerase II termination machinery (14, 34). However, although the conventional genome editing tools for *M. maripaludis* have contributed greatly to the knowledge of archaeal biology and potential biotechnological applications, further improvements are warranted to achieve faster, more efficient, and more versatile genome editing, which will enhance *M. maripaludis* as a model in archaeal biology and a chassis in archaeal biotechnology.

CRISPR-Cas9 is a next-generation platform technology that offers unprecedented robustness, speed, and efficiency in genome editing and has been proven its high values in both scientific research and biotechnological applications. Therefore, this work attempted to develop the CRISPR-Cas9 genome editing toolkit in the CO_2_-fixing methanogenic archaeon *M. maripaludis*. Through this toolkit, we have successfully and efficiently achieved the following genetic manipulations: markerless deletions of a single gene, of two genes at distant genomic loci, of an ~9-kb DNA fragment, and even of 13 genes located at three distant genomic loci, genomic modifications in the *in situ* chromosomal locations for tagging targeted genes, and mutagenesis of multiple nucleotides and even single nucleotides. Additionally, using the Cas9-based mutagenesis strategy, this work investigated the functions of transcriptional terminator and the key residues of the promoter at the *in situ* chromosomal locus of *M. maripaludis*. The robust Cas9-based genome editing toolkit developed in this work will pave the way for exploring *M. maripaludis* as a noteworthy cellular factory to facilitate fundamental biological investigations and biotechnology development of archaea.

## RESULTS

### Design of a single plasmid based CRISPR-Cas9 system in *M. maripaludis*.

**FIG 1:**
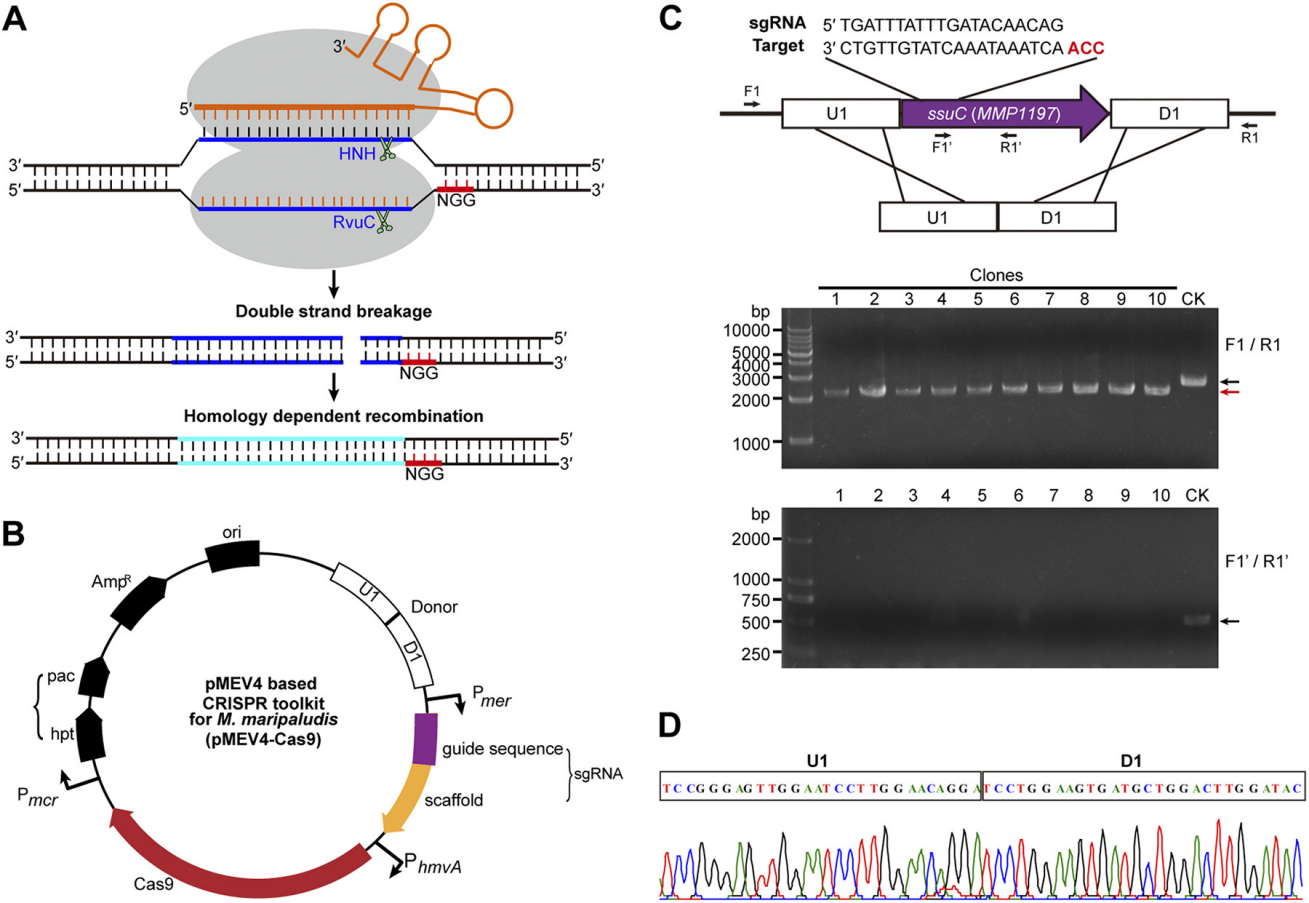
Schematic illustration of the CRISRP-Cas9 system developed in *M. maripaludis* and its application in the deletion of *MMP1197*. (A) Diagram of the Cas9-based genome editing. The Streptococcus pyogenes Cas9 and also a chimeric small guide RNA (sgRNA), which contains a 20-bp sequence derived from the host chromosome (blue) immediately upstream of a NGG PAM (red) and an 80-bp scaffold sequence (orange) to facilitate Cas9 binding, were inserted into the pMEV4 series plasmids and expressed under the respective promoters indicated in panel B. Using a donor carrying the repair templates for homologous recombination (cyan) to repair the double-strand breakages in the polyploid chromosomes, gene deletions, tagging, and multiple-/single-nucleotide mutagenesis could be generated by the Cas9-based system. HNH, histidine-asparagine-histidine endonuclease domain of Cas9. (B) Map of the pMEV4-based Cas9 editing plasmid used in *M. maripaludis*. Cas9 and sgRNA were expressed under the control of the indicated promoters. Pac, puromycin transacetylase for selection of the puromycin-resistant (Pur^r^) transformants that carry the Cas9 editing system; Hpt, hypoxanthine phosphoribosyltransferase to confer a sensitivity to the base analog 8-azahypoxanthine used in curing the Cas9 editing plasmid. (C) Knockout of *ssuC* (*MMP1197*) using the Cas9 editing system. Cas9 was guided by the sgRNA that targets the ORF internal of *ssuC* upstream of a CGG PAM (red) to bring a DSB at the *ssuC* locus and a donor containing a homologous recombinant template to delete 456 bp in the *ssuC* ORF (purple arrow) (upper portion). PCR screening of 10 selected transformants after one-step transformation and puromycin resistance selection was performed. Primers F1/R1 for specific amplifying the genome region flanking the *ssuC* ORF and F1′/R1′ for amplifying the deleted region were used to detect the deleted region residuals in the polyploid chromosomes (lower portion). Black and red arrows indicate the PCR products amplified from the wild-type (lane CK) and the mutated (lanes 1 to 10) genomes, respectively. M, double-stranded DNA (dsDNA) size marker. (D) A representative sequencing result of the PCR product amplified from the genomes of the selected transformants with *ssuC* deletions in panel C.

The shuttle expression vector pMEV4 in Escherichia coli and *M. maripaludis*, which was derived from the pAW42 vector and carried the *pac* gene for puromycin resistance as the selectable marker (27, 29, 46), was used as a backbone to harbor the two indispensable components of the CRISPR-Cas9 system: the nuclease of S. pyogenes Cas9 (SpCas9) and the “homing device” small guide RNA (sgRNA), which was constructed by fusing a *crispr* RNA (crRNA) and an associated transactivating crRNA (tracrRNA) (47, 48). SpCas9 was codon optimized and then fused downstream of P*hmvA*, a constitutive promoter commonly used in *M. maripaludis* (27, 29, 46). The sgRNA, which carries a 20-nucleotide (nt) guide sequence targeting the selected gene region and an 80-nt scaffold sequence from a fusion of crRNA and tracrRNA (Fig. 1A and B), was fused downstream of P*mer*, a constitutive promoter derived from the *mer* gene (*MMP0058*), which encodes coenzyme F_420_-dependent N^5^,N^10^-methylenetetrahydromethanopterin reductase, and the P*mer*-sgRNA was inserted upstream of the P*hmvA*-Cas9 in pMEV4. A donor sequence carrying the homologous recombinant (HR) sequence was constructed upstream of the P*mer*-sgRNA when an HR event was required. Moreover, a negative selectable marker gene, the *M. maripaludis hpt* gene, encoding hypoxanthine phosphoribosyltransferase (which confers sensitivity to the base analog 8-azahypoxanthine [49]), was introduced upstream of the *pac* gene in pMEV4 to obtain an *hpt*-*pac* cassette, encoding the counterselectable and selectable markers. The selectable marker was used to obtain transformants carrying the vector, while the counterselectable marker was then used to force the removal of the vector after the CRISPR-Cas9 editing event.

### Exploration of CRISPR-Cas9-based system for gene knockout in *M. maripaludis*.

To establish the potential application of CRISPR-Cas9 in *M. maripaludis*, soluble expression and toxicity of SpCas9 when expressed in *M. maripaludis* were first examined. Codon-optimized SpCas9 was fused downstream of P*hmvA* in the expression vector pMEV4 to obtain the plasmid pMEV4-P*hmvA*-Cas9 (referred to as pMEV401) (see Table S1 in the supplemental material), which was then transferred into *M. maripaludis* S0001, a parental strain compatible with pMEV4 (27). The band of SpCas9 at an expected molecular weight of 158 kDa was detected in the cell extract of *M. maripaludis* by Western blot assay using a commercial anti-SpCas9 antibody (Fig. S1), indicating the successful expression of SpCas9 in *M. maripaludis*. No obvious growth retardation was observed for S0001(pMEV4-SpCas9) compared with the parental strain S0001 that carries the control pMEV4 plasmid, suggesting that the expression of heterologous SpCas9 exhibits no toxicity in *M. maripaludis*.

Next, pMEV4-sgRNA-SpCas9 (referred to as pMEV402) (Table S1) was constructed to guide SpCas9 to achieve a double-strand break (DSB) at the intended target site of *ssuC*, a gene encoding the putative sulfonate transport system permease protein, which was determined to be nonessential in *M. maripaludis* (50). Vectors pMEV4, pMEV401, and pMEV402 were then separately transferred into *M. maripaludis* and the transformant counts were compared. Comparable numbers of transformants per microgram of plasmid DNA were obtained by transforming pMEV4 and pMEV401 (~7 × 10^4^ transformants), whereas only ~10 transformants was obtained when transforming pMEV402 (Fig. S2). The dramatic decrease of transformants caused by the transformation of pMEV402 compared with that of pMEV401 strongly suggests that sgRNA could guide SpCas9 to generate a lethal DSB at the target sequence in *M. maripaludis*.

Considering that the native homology-dependent recombination (HDR) machinery in *M. maripaludis* is highly efficient, a nontarget donor carrying the HR sequence with the *ssuC* open reading frame (ORF) deleted was therefore introduced to repair the lethal DSB generated by the sgRNA-SpCas9 complex. To do this, a donor, i.e., an HR arm consisting of a sequence immediately upstream (U1; ~600 bp) and another downstream (D1; ~600 bp) of the *ssuC* gene, was inserted upstream of the P*mer*-sgRNA in pMEV402 to obtain the plasmid pMEV403 (Fig. 1B). Notably, when transformed into *M. maripaludis* S0001, the donor loaded pMEV403 yielded a number of transformants (~8 × 10^4^) comparable to those of pMEV401 (Fig. S2), indicating that the donor sequence successfully repaired the chromosomal DSB generated by sgRNA-guided SpCas9 and that the transformants could contain the mutated sequence. For validation, 18 of the Pur^r^ transformants were genotyped by PCR amplification of the edited *ssuC* locus. A PCR product with the expected size (~2.3 kb) was amplified from all selected transformants using the chromosomal primers F1 and R1 (Fig. 1C), and DNA sequencing confirmed that a 437-bp deletion designed in the donor was precisely introduced into the chromosome (Fig. 1D). In support of these findings, no PCR product was amplified from the deleted chromosomal region using the primers F1′ and R1′ (Fig. 1C). Therefore, the CRISPR-Cas9 system using the HR template (donor) achieved a highly efficient and precise (100%) knockout for the 437-bp *ssuC* in 18 selected transformants. Given the extreme chromosome polyploidy of *M. maripaludis*, with up to 55 chromosome copies in the exponential phase, the Cas9-mediated genetic tool developed in this work showed impressive efficiency and precision in genome editing, which could be attributed to the robustness of Cas9-mediated DSB as well as active HDR activity in *M. maripaludis*. Moreover, the successful obtainment of the Δ*ssuC* mutant through the Cas9 system confirmed the nonessentiality of this gene in *M. maripaludis*.

### Synchronous knockout of two genes located at different genomic loci.

**FIG 2:**
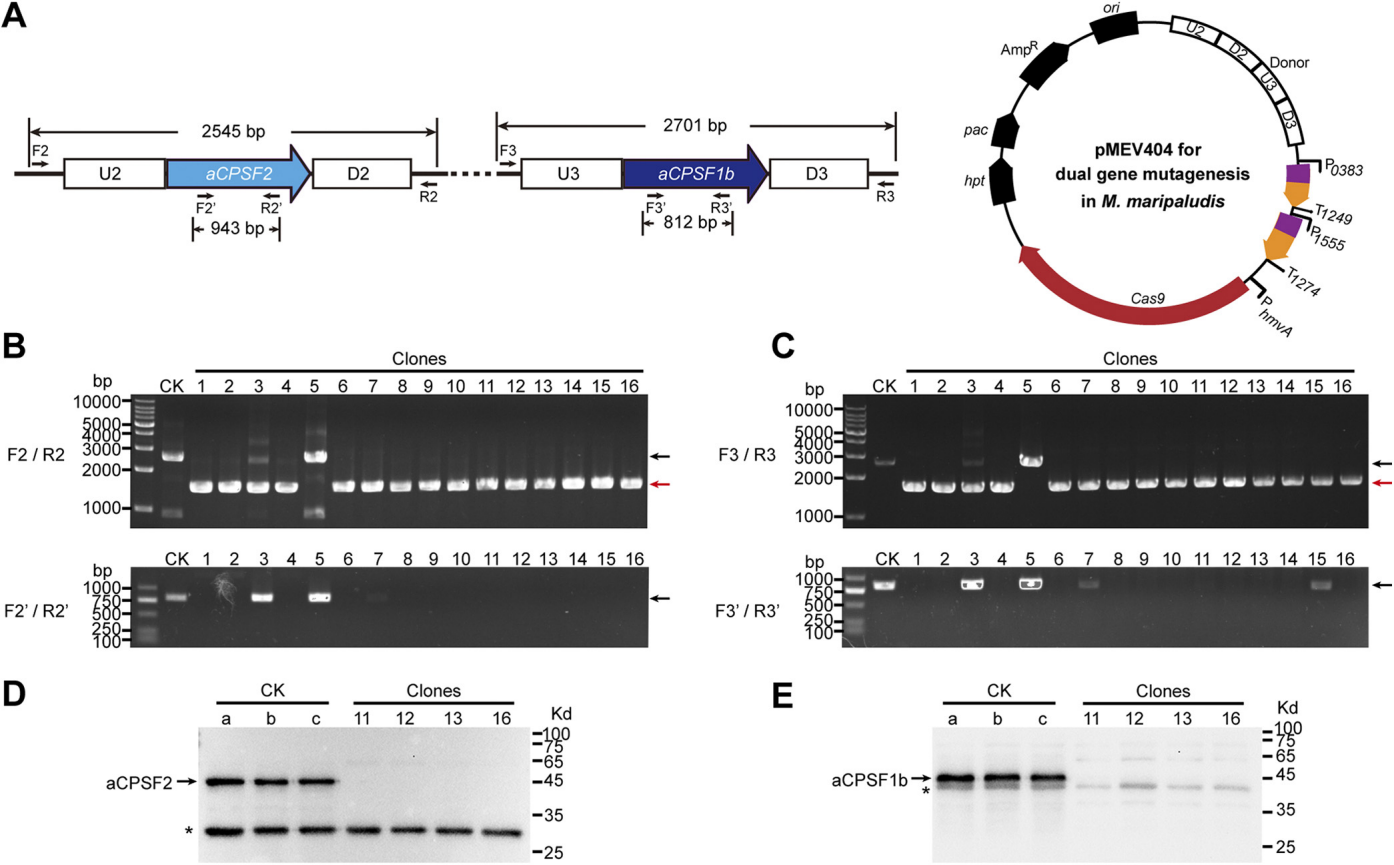
Synchronous deletion of two genes in *M. maripaludis* using the Cas9 editing system via one-step transformation. (A) Designed schematic of synchronous deletion of the genes *aCPSF2* and *aCPSF1b*, which encode two putative β-CASP family ribonucleases (left). Plasmid pMEV404 encodes two sgRNAs that match the ORF internal sequences of *aCPSF2* and *aCPSF1b* (right). The sequences upstream (U2 and U3) and downstream (D2 and D3) of each target gene were concatenated on pMEV404 to construct the recombinant donor. (B and C) Sixteen puromycin-resistant transformants were randomly selected and subjected to PCRs for detecting the knockout of *aCPSF2* (B) and *aCPSF1b* (C). The PCR primer pairs F2/R2, F2′/R2′, F3/R3, and F3′/R3′ were designed similarly to F1/R1 and F1′/R1′, as described in the legend of Fig. 1. Black and red arrows indicate the PCR products amplified from the wild-type (lane CK) and the mutated genomes (lanes 1-16), respectively. M, dsDNA size marker. (D and E) Western blot assay determined the knockout of *aCPSF2* (D) and *aCPSF1b* (E) in the selected transformants. Arrows indicate the respective protein bands of aCPSF2 and aCPSF1b through hybridization with the antibody of each protein. Asterisks indicate a band resulting from nonspecific hybridization of the polyclonal antibody. A protein ladder with molecular weights indicated is shown at the right.

Next, we tested the efficiency of the CRISPR-Cas9 system to synchronously delete two distantly located genes in *M. maripaludis*, *MMP0431* and *MMP1381*, which encode aCPSF2 and aCPSF1b, respectively. Both genes encode putative members of the β-CASP family of ribonucleases. The Cas9-carried pMEV4 plasmid was modified as pMEV404 through two steps. (i) two sgRNAs that targeted the ORF internal regions of *MMP0431* and *MMP1381* were designed. They were expressed from separate promoters, P0383 and P1555, which were derived from the highly expressed genes *MMP0383* and *MMP1555* in *M. maripaludis*, respectively (33, 35) (Fig. 2A). (ii) Two donors, each consisting of the sequences immediately upstream (U2 and U3; ~600 bp) and downstream (D2 and D3; ~600 bp) of *MMP0431* and *MMP1381*, were inserted upstream of the coding sequence of the sgRNA (Fig. 2A). The transformation efficiency of pMEV404 was comparable with that of pMEV403, and 16 clones were selected to test the knockout efficiency of *MMP0431* and *MMP1381*. Based on PCR amplification using the primer pairs F2/R2 and F3/R3 (Fig. 2B and C), the 943 bp in *aCPSF2* (*MMP0431*) and 812 bp in *aCPSF1b* (*MMP1381*) were synchronously deleted from the chromosomes of 15 out of 16 selected transformants (Fig. 2B and C). Considering the high number of polyploid chromosomes in *M. maripaludis*, to confirm the complete knockout of the two genes, we also amplified their targeted deletion regions using the primer pairs F2′/R2′ and F3′/R3′, respectively. PCR products from *aCPSF2* and *aCPSF1b* could be observed in two clones (3 and 5), and weak bands from *aCPSF1b* were observed in two additional clones (7 and 15), indicating the occurrence of heterozygous genotypes among the polyploid genomes in the four clones. Consistently, weak and large bands amplified from the wild-type chromosomes could also be observed in clones 3 and 5. Therefore, the PCRs indicated that 75% of transformants (12 of 16 clones) achieved complete knockout of the two genes from all the chromosome copies, and PCRs to amplify both outside and inside the targeted deletion regions simultaneously were required to obtain a definitive conclusion. The expression of aCPSF2 and aCPSF1b was not detected in transformants 11, 12, 13, and 16 via Western blot assay (Fig. 2D and E). Therefore, the CRISPR-Cas9 approach developed in this work can synchronously delete two genes located at different genomic loci with high efficiency and precision in the polyploid chromosomes of *M. maripaludis*. The successful deletion of the *aCPSF1b* and *aCPSF2* genes using the Cas9 system further confirmed their nonessentiality in *M. maripaludis*, as indicated by the transposon mutation system (50).

### CRISPR-Cas9 facilitates the deletion of large DNA fragments in *M. maripaludis*.

**FIG 3:**
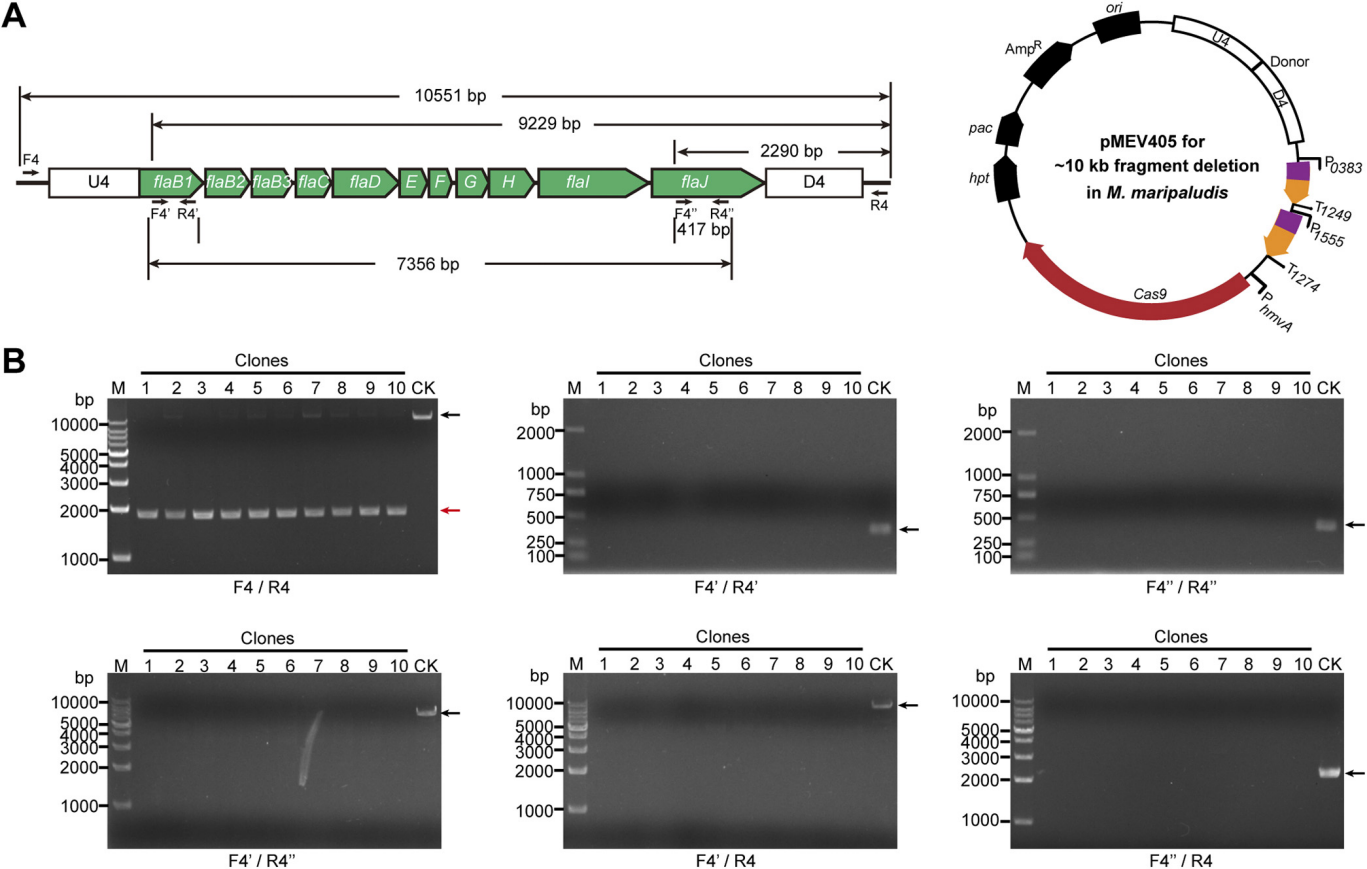
Deletion of an ~9-kb DNA fragment in *M. maripaludis* using the Cas9 editing system by one-step transformation. (A) Schematic of the plasmid designed for deleting the ~9-kb *fla* operon that synthesizes archaella (left). Plasmid pMEV405 encodes two sgRNAs that match the first (*MMP1166*) and the last (*MMP1176*) genes in the *fla* operon. The sequences upstream (U4) and downstream (D4) of the *fla* operon were concatenated on pMEV405 to provide the recombinant donor. (B) Ten puromycin-resistant transformants were randomly selected for PCRs using the primer pairs F4/R4, F4′/R4′, F4″/R4″, F4′/R4″, F4′/R4, and F4″/R4, which target the regions indicated in panel A. Black and red arrows indicate the PCR products amplified from the wild-type (lane CK) and mutated genomes (lanes 1-10), respectively. M, dsDNA size marker.

To explore the knockout efficacy of large DNA fragments in *M. maripaludis*, the *fla* operon at a length of 9,043 bp was selected (Fig. 3A). This operon contains 11 coding genes involved in the synthesis and assembly of archaella, the swimming organelles found in archaea (36, 51, 52), and has been determined to be nonessential in *M. maripaludis* (50). The Cas9-carried pMEV4 was modified to create pMEV405 in the following process: (i) two sgRNAs, targeting the ORF internal region of *flaB1* and *flaJ*, were expressed by fusing downstream of the promoters of P0383 and P1555, respectively; (ii) a donor, consisting of the sequences immediately upstream (U4; ~600 bp) of the coding regions of *flaB1* and downstream (D4; ~600 bp) of *flaJ*, was inserted upstream of the sgRNA (Fig. 3A). After puromycin resistance selection, 10 transformants were selected for PCRs. Using the primers F4/R4, instead of a 10,551-bp PCR product generated from the parental S0001 strain, shortened PCR products were obtained from all 10 transformants (Fig. 3B, F4/R4), suggesting the successful deletion of the entire *fla* operon. Faint PCR products comparable in length to those amplified from S0001 could be observed in four clones (clones 2, 5, 7, and 8). To discern whether these large, faint PCR products were derived from the *fla* operon remaining on a few genomes of the polyploid cells or the nonspecific amplification of F4/R4, five additional primer pairs were used for further tests. The primer pairs F4′/R4′ and F4″/R4″ can amplify the 377-bp and 417-bp internal regions of *flaB1* and *flaJ*, respectively (Fig. 3A). However, neither PCR product was detected from any of the 10 transformants (Fig. 3B). The primer pair F4′/R″ can amplify the 7356-bp region of *flaB1* to *flaJ* but failed to yield a product for all 10 transformants (Fig. 3A and B). Similarly, no products at a length of 9,229 bp or 2,290 bp were amplified from the 10 transformants by primers F4′/R4 and F4″/R4, which target *flaB1* and *flaJ*, respectively, and R4 outside the *fla* operon (Fig. 3A and B). Therefore, the PCR assay verified the complete deletion of the entire *fla* operon on the polyploid chromosomes in the 10 randomly selected transformants, demonstrating that the CRISPR Cas9-based genome editing method is capable of the precise deletion of an ~9-kb fragment with high efficiency (100%) in *M. maripaludis*.

### Synchronous deletion of 13 genes in different genomic locations of *M. maripaludis*.

**FIG 4:**
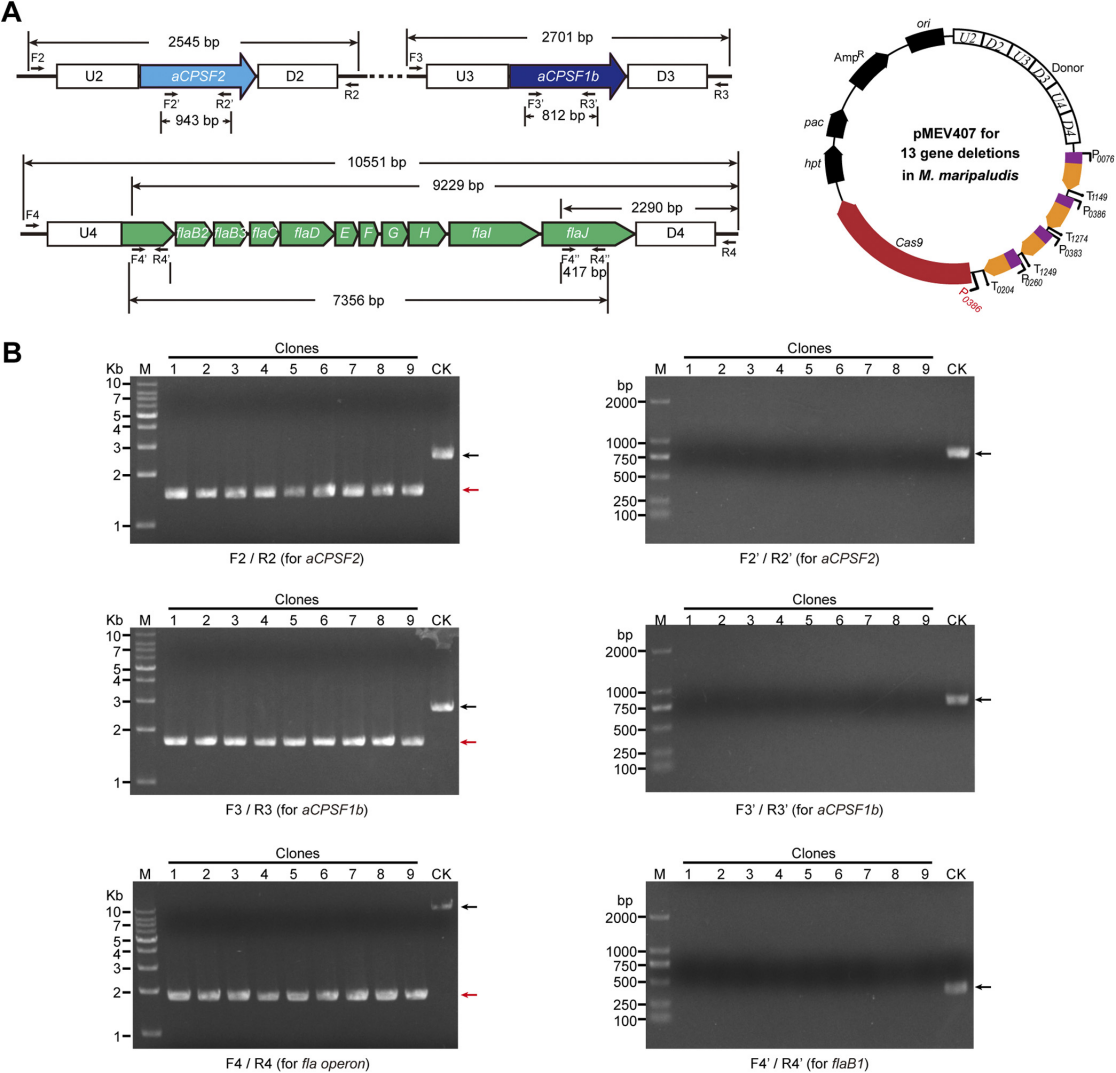
Synchronous deletion of multiple (13) genes substituted in three genomic loci in *M. maripaludis* using the Cas9 editing system. (A) Schematic of the plasmid designed for the synchronous deletion of 13 genes consisting of the *fla* operon genes (*MMP1666* to *MMP1676*) and *aCPSF2* (*MMP0431*) and *aCPSF1b* (*MMP1381*). Plasmid pMEV407 encodes four sgRNAs that match *aCPSF2*, *aCPSF1b*, *flaB1* (*MMP1666*), and *flaJ* (*MMP1676*) (right). They were expressed under the control of the indicated promoters, which were derived from the highly expressed genes in *M. maripaludis*. The sequences upstream (U2, U3, and U4) and downstream (D2, D3, and D4) of each gene were concatenated on pMEV407 to provide the recombinant donor. Distinctly, the Cas9 in pMEV407 was expressed under the control of promoter P0386 from the highly expressed gene *MMP0386*. (B) Nine puromycin-resistant transformants were randomly selected and subjected to PCR assay. Primer pairs F2/R2, F2′/R2′, F3/R3, F3′/R3′, F4/R4, and F4′/R4′ were used to detect the knockout of *aCPSF2*, *aCPSF1b*, and the *fla* operon genes, respectively. Black and red arrows indicate the PCR products amplified from the wild-type (lane CK) and the mutated genomes (lanes 1-9), respectively. M, dsDNA size marker.

Given the complexity of intracellular metabolic networks, synchronous modification of multiple genes is frequently required in basic and applied research. Based on the robustness and functional modularity of CRISPR systems, we explored the multiplexing capability of the Cas9-based genetic tool to synchronously knock out 13 genes located in three different genomic loci, a large DNA fragment of the *fla* operon and two other distantly distributed genes (*aCPSF2* and *aCPSF1b*) in *M. maripaludis*. To achieve this, the Cas9-carried pMEV4 plasmid was modified to create pMEV406 as follows: (i) four sgRNAs, each targeting the internal regions of *aCPSF2* (*MMP0431*), *aCPSF1b* (*MMP1381*), *flaB1* (*MMP1666*), and *flaJ* (*MMP1676*), were transcribed under four independent promoters and terminators derived from the indicated genes shown in Fig. 4A according to our previous works (33, 35); (ii) the donor of U4-D4 shown in Fig. 3 was fused downstream of the two donors of U2-D2 and U3-D3 shown in Fig. 2 and inserted upstream of the four sgRNA elements (Fig. 4A). After being transferred into strain S0001 and selected by puromycin resistance, nine transformants were randomly selected for PCRs by using two sets of primer pairs to amplify the external and the internal targeted regions of each of *aCPSF2*, *aCPSF1b*, and the *fla* operon. The PCR assay determined the 100% deletion of 943 bp in *aCPSF2* (*MMP0431*) in the nine selected transformants (Fig. S3, F2/R2 and F2′/R2′), while the deletion of 812 bp in *aCPSF1b* (*MMP1381*) was achieved in only one of the nine (<11.1%) selected transformants (Fig. S3, F3/R3 and F3′/R3′), which implied that the editing efficacy of *MMP1381*-sgRNA was lower than that of *MMP0431*-sgRNA. The deletion of the ~9-kb *fla* operon was also detected. Using the primers F4/R4, amplifying the external genomic region of the *fla* operon, shortened PCR products were obtained in seven of the nine transformants, indicating the expected deletion of the *fla* operon, while large products in comparable length to the control amplified from the wild-type *fla* operon were also detected (Fig. S3, F4/R4). This indicated the incomplete editing of the *fla* operon from the polyploid cells, which was confirmed by the PCR result of F4′/R4′ for amplifying the internal region of *flaB1* (Fig. S3, F4′/R4′). Therefore, these results indicated that when using plasmid pMEV406, only *aCPSF2* was deleted with high efficiency (100%), but not the other two targeted regions (*aCPSF1b* and *fla* operon), possibly because the sgRNA of *aCPSF2* had a higher targeting efficiency than the other two.

The incomplete synchronous deletion of *aCPSF1b* and the ~9-kb *fla* operon on all the polyploid chromosomes by pMEV406 inspired us to consider possible reasons. That the expected deletions of *aCPSF1b* and the *fla* operon were generated on some of the polyploid chromosomes indicated that the sgRNAs for them were effective, while the incomplete synchronous deletions for the two DNA regions implied a possibility of incomplete DSBs on all of the polyploid chromosomes. This could have been caused by the inadequate expression of SpCas9 but not by the expression of sgRNAs, which were all expressed under the control of promoters of highly expressed genes (33, 35). SpCas9 in pMEV406 was expressed under the control of promoter P*hmvA*, a conventionally used promoter in *M. maripaludis* (29, 46), and its expression strength was recently determined to be moderate (53). Therefore, we used promoter P0386 derived from the highly expressed *MMP0386* to replace P*hmvA* and construct plasmid pMEV407 (Fig. 4A). Western blot assay demonstrated that the expression of SpCas9 in pMEV407 was clearly improved, more than 3-fold, compared to that in pMEV406 (Fig. S4). Upon transforming pMEV407 into strain S0001 and conducting puromycin resistance selection, 10 transformants were randomly selected for PCR assay as described above. It was noteworthy that the PCR results for amplifying both the external and the internal targeted editing regions using the sets of primer pairs described above clearly demonstrated the synchronous deletion of *aCPSF2*, *aCPSF1b*, and the *fla* operon on all of the polyploid chromosomes of the 10 selected transformants (100% editing efficiency for the three genomic loci) (Fig. 4). Therefore, by improving the expression of SpCas9 in pMEV407 using a stronger promoter, high efficiency and precision (100%) for the synchronous deletion of multiple genes, even for 13 genes located on three genomic loci, could be achieved by one-step transformation in *M. maripaludis* using the CRISPR-Cas9 genetic tool developed in this study.

### Cas9-based *in situ* tagging of a chromosomal gene.

**FIG 5:**
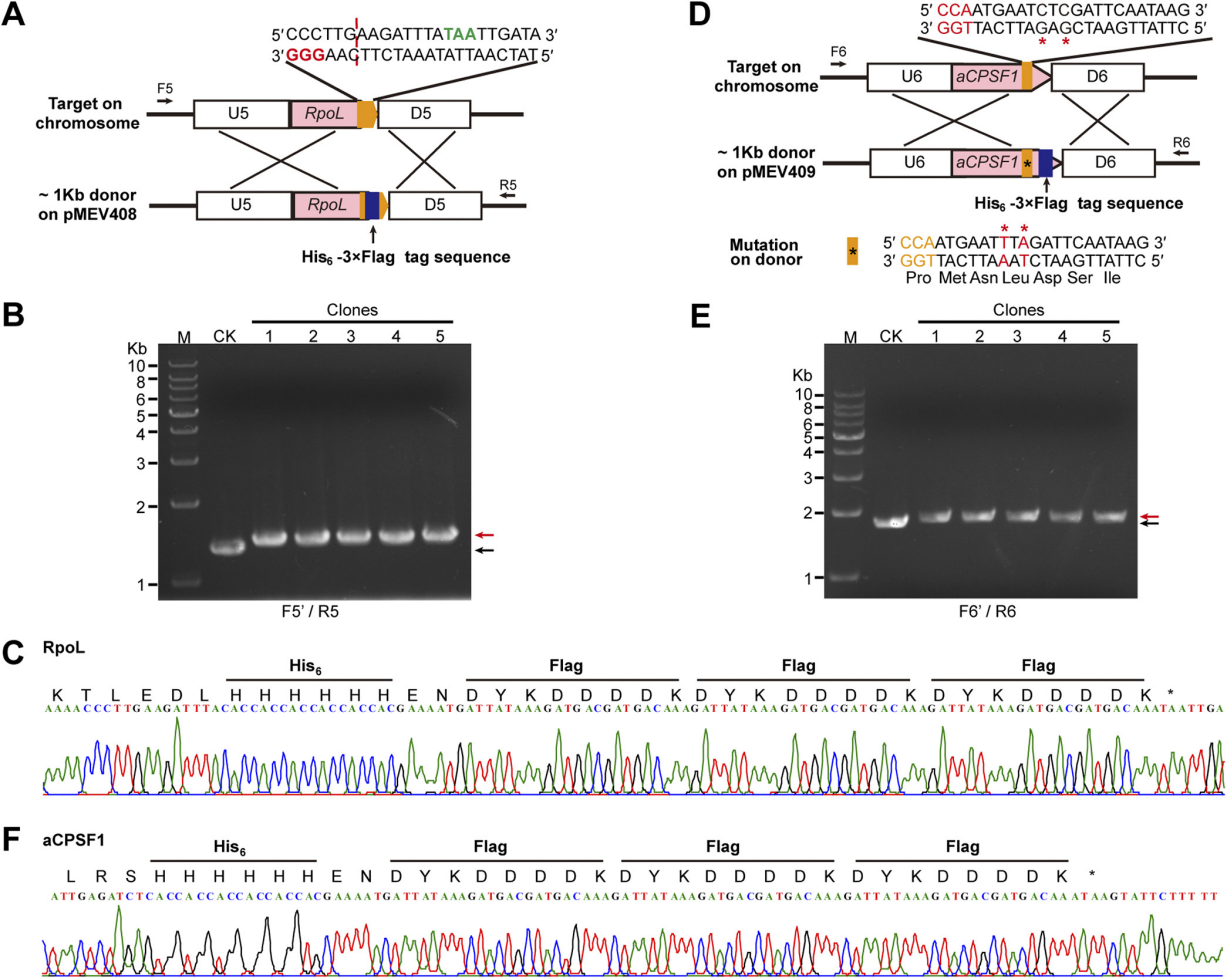
Cas9-based *in situ* tagging of the essential genes in *M. maripaludis*. (A and D) Schematics showing the *in situ* tagging at two genes encoding the RNA polymerase subunit L (RpoL) (A) and the transcription termination factor aCPSF1 (D). The pMEV4 plasmid was used to express sgRNA targeting the C terminus of RpoL or aCPSF1 (orange blocks); the donor sequences contain RpoL and aCPSF1 with the C terminus fused with His_6_-3×Flag tag (blue block). The PAM nucleotides are shown in red letters. (B and E) Five randomly selected transformants were screened using PCR with chromosome-specific primers F5/R5 or F6/R6 as depicted in panels A and D. Black and red arrows indicate the PCR products amplified from the wild-type and mutated genomes, respectively. M, dsDNA size marker. (C and F) DNA sequences of representative PCR products amplified using F5/R5 and F6/R6, depicted in panels A and D, from the 5 mutants.

*In situ* tagging is necessary for investigating the cellular functions of genes under physiological conditions, where the studied gene is expressed at its *in situ* condition. Thus, we explored Cas9-based genome editing method to achieve the *in situ* tagging of two essential genes, those encoding the RNA polymerase subunit L (RpoL) and the recently identified general transcription termination factor aCPSF1 (33, 34). Plasmid pMEV4 was modified by changing both the sgRNA and the donor sequences. For the tagging of RhoL, an sgRNA targeting the sequence spanning the stop codon TAA of *rpoL* was chosen to be expressed in the pMEV4 plasmid, and an ~1-kb sequence that flanks the sgRNA targeting region by each ~500 bp up- and downstream of the TAA of *rpoL* was amplified to generate a donor (Fig. 5A). A 96-bp sequence encoding 6×His (His_6_) and 3×Flag tags with a short linker (2 amino acids) between the two tags was inserted upstream of the TAA of *rpoL* into the donor (Fig. 5A). Plasmid pMEV408 was consequently constructed and transferred into *M. maripaludis*. After puromycin resistance selection, transformants were randomly selected for PCR assay using two chromosome-specific primers, F5 and R5 (Fig. 5A), and slightly lagged PCR bands compared to those amplified from the wild-type chromosome were amplified (Fig. 5B). DNA sequencing of the PCR products further verified the correct tagging (Fig. 5C). This demonstrated that the His_6_-3×Flag tag sequence was successfully inserted into the targeted chromosomal locus using the CRISPR-Cas9 strategy.

However, as no protospacer-adjacent motif (PAM) sequence (NGG) was present proximate to the stop codon of aCPSF1, an sgRNA targeting the sequence 7 bp upstream of the stop codon TAA was selected (Fig. 5D). The donor DNA contained the same sequence as well, and the tag sequence inserted upstream of the TAA of aCPSF1 would not affect the sgRNA targeting the donor. Therefore, the sgRNA would inevitably target the donor DNA where Cas9 had created a DSB and thus impaired the intended HDR, making it difficult to obtain the positive tagged transformants. Consistently, after the first-round transformation, no tagged transformants were obtained. To avoid the cutting of Cas9 on the donor and maintain the same coding sequence, we introduced two point mutations at the sgRNA targeting sequence in the donor, which resulted in a synonymous codon mutation from TTA to AAT coding for a Leu residue (Fig. 5D). After the second-round transformation, five randomly selected transformants were tested by PCR using the chromosome-specific primer pair F6/R6 (Fig. 5E), followed by DNA sequencing of the PCR products (Fig. 5F). The assays verified that the His_6_-3×Flag sequence was successfully tagged into the C terminus of aCPSF1.

Together, these experiments demonstrated that regardless of the presence or absence of a PAM sequence at the N or C terminus, by avoiding the sgRNA targeting at the donor using the strategy used in *rpoL* and that used in *aCPSF1* by introducing the synonymous codon mutation into the donor (Fig. 5), the *in situ* tagging enabled by the CRISPR-Cas9 system could be theoretically applicable to all genes in *M. maripaludis*. Moreover, the experiments in this study have indicated that only two nucleotide mutations in the sgRNA targeting sequence can successfully prevent the sgRNA from guiding SpCas9 to cut the donor, therefore demonstrating that the Cas9 editing system can specifically discriminate two nucleotide differences to achieve precise editing.

### Cas9-based *in situ* mutagenesis of multiple nucleotides in a transcription terminator.

**FIG 6:**
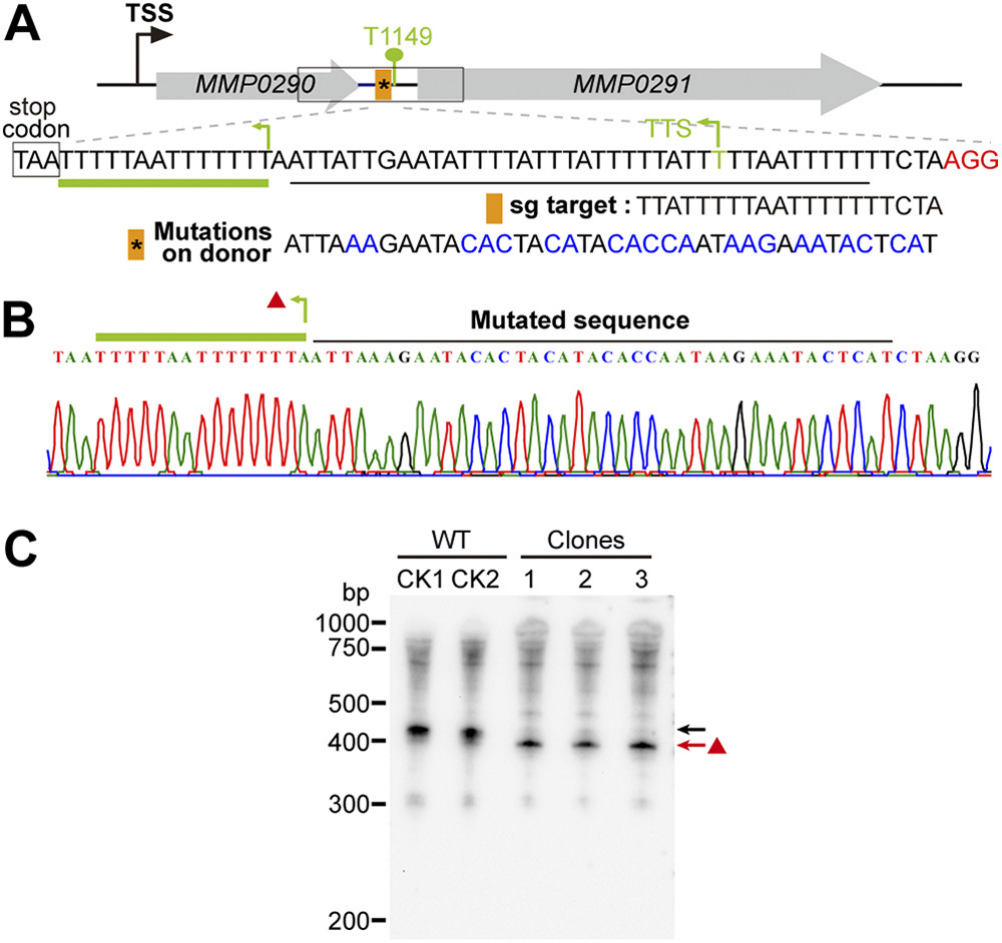
Cas9-based *in situ* multinucleotide mutagenesis to determine the key residues in a terminator of *M. maripaludis*. (A) Design schematic showing the *in situ* mutagenesis of multiple nucleotides in a terminator identified by Term-seq previously (33, 34). An sgRNA that targets the terminator region (orange bar with the asterisk indicating the nucleotide mutation) was expressed, and the multiple nucleotides in blue letters that were designed to replace the U-rich sequence of the terminator were introduced into the donor sequence at the pMEV4 plasmids. The PAM sequence is shown in red letters. TSS, transcription start site. (B) Five randomly selected transformants were screened via PCR using the primers F7/R7 depicted in panel A. Nucleotide mutations were further validated by DNA sequencing. The red triangle indicates a U-rich sequence present upstream, a potential new terminator of *MMP0290*. (C) Northern blotting detected the transcript of *MMP0290* in the wild-type (WT) (black arrow) and the multinucleotide-mutated transformants (clones 1-3) (red arrow) on the primary terminator. The red arrow points to a short transcript detected in the terminator mutants, which appeared to terminate at the upstream uridine-rich terminator sequence (green bent arrow in panel B).

Point mutagenesis is frequently required in the evaluation of the key amino acids or nucleotides in gene/protein functions. An *in situ* strategy for this process without changing the copy number or the encoding contexts of the studied gene would be optimum. Consequently, we further evaluated the ability of the CRISPR Cas9 system to generate nucleotide substitutions in a transcriptional terminator (Fig. 6A), a featured U-tract sequence upstream of the identified transcription termination site (TTS) of *MMP0290* (33, 34). To introduce point mutations in this terminator, we modified the Cas9-carried pMEV4 by expressing an sgRNA that targeted a 20-nt sequence flanking the TTS of *MMP0290* and inserting an ~1-kb DNA sequence flanking the TTS and with it in the center as the donor. Then T-to-A or -C mutations were introduced into the donor as shown in Fig. 6A and the constructed plasmid pMEV410 was transformed into *M. maripaludis*. Puromycin-resistant transformants were subjected to PCR and DNA sequencing. Mutated nucleotides instead of the wild-type terminator sequence were found in the PCR products from five randomly selected transformants (Fig. 6B), confirming that the CRISPR-Cas9 genome editing tool generated a successful point mutation at the *MMP0290* terminator in the polyploid chromosomes of *M. maripaludis* with high efficiency (100%).

Then we evaluated the effects of terminator mutation on the transcription of *MMP0290*. As shown in Fig. 6C, the Northern blot assay detected, in contrast to a >400-bp transcript of *MMP0290* in the parental strain S0001, a slightly shorter transcript in all the *MMP0290* terminator mutants, implying that an early termination of *MMP0290* occurred in the mutants. Through further inspection of the sequence upstream of the *MMP0290* TTS, another two U-rich tracts very similar to the terminator feature identified in *M. maripaludis* (33, 34) were found, implying a potential upstream termination site that could be masked by the secondary structure of the RNA in the parent strain. This indicates that the Cas9 system not only is a robust genetic tool for *in situ* point mutagenesis but also may facilitate the identification of new genetic elements, such as the early terminator found in this study.

### Cas9-based *in situ* mutagenesis of a single nucleotide to evaluate the key element of the promoter.

**FIG 7:**
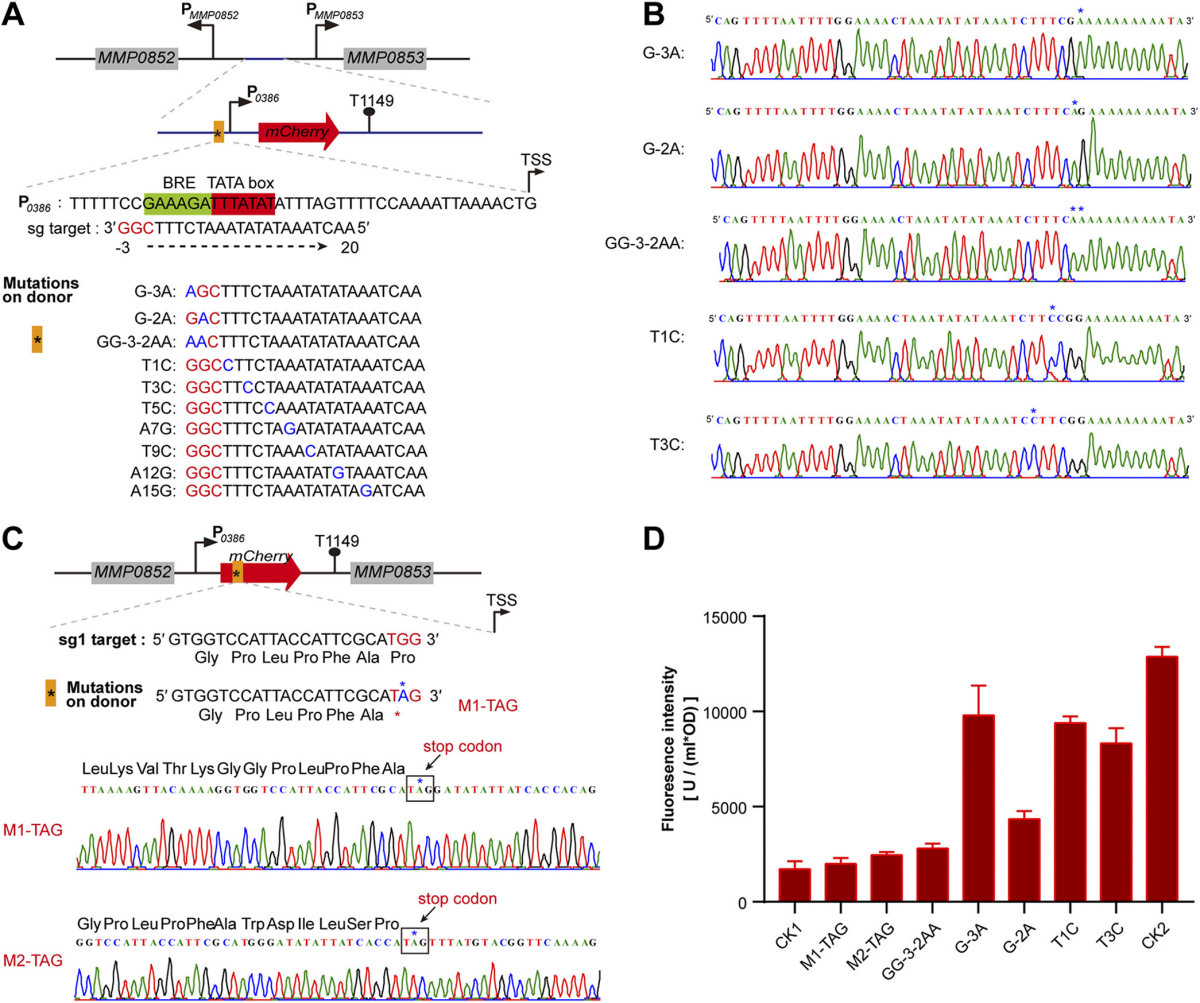
Cas9-based *in situ* mutagenesis of single nucleotides for determining key residues in promoter or inactivating the expression of the target gene in *M. maripaludis*. (A) Design schematic showing the *in situ* mutagenesis of single-nucleotide substitution in P0386, the promoter of *MMP0386*, which is fused with the reporter mCherry gene to obtain P0386-*mCherry*. P0386-*mCherry* was inserted in the intergenic region between *MMP0852* and *MMP08530* as in our previous study (34). An sgRNA targeting the promoter region (orange block) was expressed and the substitution of the single nucleotide (blue letters) to mutate the key residues of the promoter was introduced into the donor sequence on the pMEV4 plasmids. PAM nucleotides are shown in red. (B) Five randomly selected transformants were screened by PCRs, and the nucleotide mutations were further validated by DNA sequencing. (C) Design schematic of introducing a stop codon into the mCherry gene via Cas9-based *in situ* single-nucleotide substitution. An sgRNA targeting the ORF region (orange block) was expressed and the single-nucleotide substitutions (indicated by the asterisk) to introduce stop codons were generated in the donor sequence on the pMEV4 plasmids. PAM nucleotides are shown in red. Five randomly selected transformants were screened using PCRs, and the nucleotide mutations were further validated by DNA sequencing. (D) Fluorescence intensities of mCherry were assayed to determine the effects on the expression of mCherry of the single-nucleotide mutagenesis in the promoter and the introduction of a stop codon. CK1, CK2, and other symbols on the *x* axis indicate the parental strain S0001, S0001 carrying P0386-*mCherry*, and the mutation strains obtained as shown in panels A and C. Fluorescence intensities of mCherry were assayed in triplicate cultures, and averages and standard deviations are shown.

Next, we explored the application of the Cas9-based system in the *in situ* mutagenesis of a single nucleotide in *M. maripaludis* for evaluating key nucleotides of a promoter. First, an mCherry reporter gene for fluorescence monitoring was fused downstream of the promoter P0386. It was then inserted into the intergenic region (IGR) of *MMP0852* and *MMP0853* (Fig. 7A) as described previously (34). Coincidently, an sgRNA sequence targeting the promoter region, TATA box, and BRE (transcription factor B [TFB] recognition element) could be chosen to construct the Cas9-based pMEV4 plasmid. By adopting the conventional numbering rule, the three PAM nucleotides were designated positions −1, −2, and −3, with −1 closest to the target sequence, and the sgRNA targeting nucleotides were referred to as 1, 2, 3, and so on, with 1 closest to PAM. An ~1-kb sequence by each ~500 bp up- and downstream of the sgRNA targeting region was amplified as the donor and inserted upstream of the sgRNA element in the Cas9-carried pMEV4. Next, a series of single-nucleotide substitutions was introduced into the sgRNA targeting sequence in the donor at positions −3, −2, 1, 3, 5, 7, 9, 12, and 15 and a synchronous substitution of two nucleotides at positions −3 and −2 (GG-3-2AA). Interestingly, after the selection of transformants and PCR verification, the double-nucleotide mutation GG-3-2AA and the single-nucleotide substitutions G-3A, G-2A, and T1C could be obtained at high efficiencies (75% to 100%); T3C was obtained at a lower efficiency (25% to 50%), and T5C, A7G, T9C, A12G, and A15G could not be obtained. This result suggests that a donor containing a point mutation at position 5, 7, 9, 12, or 15 could still be recognized and cleaved by CRISPR-Cas9 and thus could not serve as an effective repair template. Each five randomly selected transformants of the mutants G-3A, G-2A, T1C, T3C, and GG-3-2AA were examined by PCR using chromosome-specific primers for amplifying the targeting locus (Fig. 7A) and DNA sequencing. The result verified the successful mutations of the single or double nucleotides in all the selected transformants (Fig. 7B).

Then the effects of these single- or double-nucleotide mutations on the activity of P0386 were evaluated by monitoring mCherry fluorescence. The result demonstrated that the four single-nucleotide mutations in the BRE region all decreased the promoter activity and the G-2A mutation displayed a dramatic decrease, while the double-nucleotide mutation (GG-3-2AA) almost completely eliminated the promoter activity of P0386 (Fig. 7D). These experiments not only demonstrated the importance of the BRE nucleotides in promoter activity but also indicated that using the CRISPR-Cas9 strategy, the *in situ* mutation of a single nucleotide could be introduced into the polyploid chromosomes of *M. maripaludis* in one step, while high mutation efficiency may be restricted in the region proximal to PAM, e.g., −3, −2, 1, and 3.

### Inactivation of coding genes by introducing a stop codon through Cas9-based single-nucleotide mutagenesis.

Next, the mutation of a single nucleotide using the Cas9-based strategy was applied to introduce a stop codon into a gene and so suppress its expression in *M. maripaludis*. Two sgRNAs (sg1 and sg2) that targeted the 5′ terminus of the mCherry ORF were separately inserted into the Cas9-carried pMEV4 downstream of P*mer*, and a donor of ~1 kb with each ~500 bp up- and downstream of the sgRNA targeting sequence was inserted upstream of the sgRNA. Then a single-nucleotide substitution was introduced into the sgRNA (sg1 or sg2) targeting sequences in the donors, with a stop codon introduced into the two separate donors by mutating the −2 position of sg1 (TGG to TAG by G-2A, M1-TAG) and the 1 position of sg2 (CAG to TAG by C1T, M2-TAG) (Fig. 7C). After transformation of *M. maripaludis*, five transformants were randomly selected for PCR and DNA sequencing. The results verified that the single-nucleotide substitutions had all successfully been generated and the stop codon had been introduced into the mCherry gene at the expected sites in M1-TAG and M2-TAG (Fig. 7C). Fluorescence assay determined that the expression of mCherry was decreased almost to the background value of the parental strain S0001 (Fig. 7D). This demonstrated that the introduction of the stop codon into mCherry had successfully aborted its expression. Therefore, stop codon introduction via the Cas9-based single-nucleotide mutation strategy provides another genome editing approach for suppressing gene expression.

### Curing of the Cas9-carried pMEV4 plasmid for a new round of genome editing.

In order to successively edit a secondary genome locus in the primary mutant or for other needs, the Cas9 editing machinery should be cured from the initial gene-edited strain prior to the second round of editing. To achieve this goal, a counterselection marker, *hpt*, which was inserted into pMEV4 in the present study, was examined to facilitate the curing. The *hpt* marker confers sensitivity to the base analog 8-azahypoxanthine in the parental strain S0001, which is a Δ*hpt* mutant (49). To achieve the plasmid curing, the transformants with puromycin resistance were subcultured for four or five generations in liquid medium without puromycin, and then the culture was plated onto a solid counterselection medium containing 8-azahypoxanthine to obtain the 8-azahypoxanthine resistant transformants. The 8-azahypoxanthine resistant isolates were found to be incapable of growing in puromycin containing medium (Fig. S5A), and PCR using the plasmid-specific primers did not amplify the pMEV4 sequence, confirming the complete curing of the plasmid in these isolates (Fig. S5B) and making the next genome editing action possible. This suggests that through using the counterselection marker to cure the used Cas9-carried vectors, multiple rounds of genome editing can be readily achieved.

## DISCUSSION

In this study, we developed the CRISPR-Cas9 toolkit in *M. maripaludis*, which not only exhibits high efficiency in genome editing in the polyploid chromosomes of this archaeon but also achieves a variety of genetic manipulations. Using the CRIPSR-Cas9 toolkit developed in this work, the following genetic manipulations have been successfully and efficiently achieved: markerless knockout of a single gene (Fig. 1) and two genes at distant genomic loci (Fig. 2); deletion of an ~9-kb DNA fragment (Fig. 3) and even synchronous deletion of 13 genes located at three genomic loci (Fig. 4); *in situ* genomic modification for tagging target genes (Fig. 5); and *in situ* multiple-nucleotide (Fig. 6) and even single-nucleotide (Fig. 7) mutagenesis. Moreover, using Cas9-based nucleotide mutagenesis, the functions of the transcriptional terminator and the key residues of the promoter were investigated at an *in situ* chromosomal locus (Fig. 6 and 7), and stop codon was introduced into a target gene to provide an alternative approach for suppressing gene expression in *M. maripaludis* (Fig. 7C and D). Additionally, using counterselectable markers, the Cas9-carried editing machinery could be cured from the edited cells, making multiple rounds of genome editing in one cell possible. Therefore, the robust Cas9-based genome editing toolkit developed in this work is a major step in using *M. maripaludis* as a workhorse or cellular factory, like E. coli and Saccharomyces cerevisiae, for research on the fundamental biology and the biotechnology development of archaea.

It is worth noting that given the high polyploidy in *M. maripaludis*, with up to ~55 genome copies per cell, 55 chromosomal events may be needed per locus per DSB or HDR operation. Thus, the synchronous deletion of 13 genes at three genomic loci may require a total of ~330 chromosomal breakage-repair events. To achieve these ~330 events, enhanced Cas9 expression by using a stronger promoter was essential in addition to the successful expression of four sgRNAs (Fig. 4 and Fig. S3 and S4). This is a substantial improvement upon currently available tools with which only one genomic locus can be edited per round of genetic manipulation (29, 31, 49). This is undoubtably laborious and time-consuming, especially in the traditional markerless gene editing, which conventionally needs two rounds of resistance marker selections, one for positive and the other for negative selection, and takes at least 7 to 8 weeks for editing only one genomic locus. The Cas9-based toolkit developed in this study saves much time and can edit multiple genomic loci synchronously through only one-step transformation (2 to 3 weeks). Therefore, the labor and time savings and the robustness of the Cas9-bsed genome editing for synchronously editing multiplex genes are great improvements on the conventional genetic strategy in *M. maripaludis*.

Given the complexity of intracellular metabolic networks, the Cas9 toolkit developed in this work will broaden the applications of *M. maripaludis* in both basic and applied studies when it is necessary to engineer multiple genes across different loci simultaneously. Theoretically, introduction of *n* sgRNAs can target *n* genomic loci to achieve the gene deletion or replacement mutagenesis. For example, by rationally designing the genetic elements, as many as 22 sgRNAs were stably coexpressed with long sgRNA arrays to simultaneously repress up to 13 genes located in different genomic loci in E. coli (54). Therefore, the versatile CRISPR-Cas9 toolkit developed in *M. maripaludis* in this work, especially the robust (markerless) multiplex gene editing tool, has endowed the capability and convenience to regulate many endogenous genes at the same time to trigger changes in cell state or metabolic flux, to study complex gene regulatory networks or to modify the metabolic pathways for engineering the production of high-value metabolites in both basic research and biotechnology application. Considering the natural advantages of this archaeal species, that *M. maripaludis* not only is a representative CO_2_-fixing and methanogenic archaeon but also possesses the potential of inexpensively producing high-value biochemical products, such as hydrogen, methanol, isoprenoids, and coenzymes (25), the Cas9 toolkit developed in this work will greatly facilitate engineering of key metabolic pathways to explore the biotechnology value of *M. maripaludis*.

It is noteworthy that the high genome editing efficiency of the Cas9 system has been achieved in *M. maripaludis*, an archaeon possessing a remarkable polyploid genome, with up to ~55 and 30 copies per cell in the exponential and stationary phases, respectively. These chromosomal copies are even higher than those in halobacteria, another euryarchaeal group known to possess polyploid chromosomes, with 15 to 25 copies reported for some halobacterial species (55, 56). Polyploid cells have been hypothesized to hinder precise genome editing through harnessing the native CRISPR-Cas 1b type in the halobacterial species Haloarcula hispanica (57). This polyploidy state indeed made it intrinsically difficult to achieve complete genome editing across all the 30 to 55 genome copies in *M. maripaludis*. Although the toolkit developed in this work achieved 100% efficiency in editing one locus from all the chromosomal copies (Fig. 1), the efficiency dropped to 75% when two loci were edited (Fig. 2), and the toolkit ultimately failed to perform complete editing on three loci (Fig. S3). However, the editing efficiency returned to 100% for the editing of three loci when Cas9 expression was enhanced under the control of a stronger promoter (Fig. 4), illustrating the power of this toolkit in achieving complete editing across multiple loci of polyploid chromosomes. Consequently, the highly efficient genome editing ability of the CRISPR-Cas9 toolkit developed in this study has boosted the speed and flexibility of biotechnological design and remodeling the genetic information and therefore promotes the value of *M. maripaludis* to be used as an archaeal chassis for biotechnological development, particularly for CO_2_-based remodeling.

The versatile utility of this toolkit is further demonstrated by its ability to achieve efficient and precise gene tagging and point mutagenesis, which have not been demonstrated before in *M. maripaludis*. Traditional *in situ* gene tagging using the conventional HR technique dependent on resistance genes is cumbersome and time-consuming, especially for genes organized in an operon, as the introduction of tags by resistance genes could generate polarity effects on the downstream genes, which would be lethal if they are essential genes. The Cas9-based tagging strategy surmounts these barriers with its editing precision, and no resistance gene is concomitantly introduced. However, researchers should be aware when using the Cas9-based tagging method, an sgRNA needs to be designed dependent on the occurrence of the NGG PAM sequence in the target tagging region, usually at the N or C terminus. However, when an NGG is not present in the intended tagging region, the tag sequence insertion of the donor DNA does not eliminate the target sequence and self-cutting of the donor DNA by sgRNA-guided Cas9 occurs (Fig. 5). To circumvent this issue, two point mutations resulting in synonymous mutations can be introduced in the sgRNA targeting sequence in the donor. This not only avoids self-cutting but also maintains the same amino acid sequence. This method can serve as a general method for gene tagging regardless of the position of NGG. Thus, the tagging strategy explored in this work (Fig. 5) provides an effective solution for possible tagging situations, and using the solution explored in this work, researchers can generate tagging for any gene in *M. maripaludis*. This solution also has reference value for other prokaryotic organisms. Moreover, the ability of two point mutations in the donor sequence to prevent the Cas9 cutting of the donor DNA (Fig. 5) also experimentally confirms the editing precision of this toolkit, as the Cas9 system could precisely differentiate two very similar sequences. Additionally, based on the multiple-gene knockout strategy explored in this work using four-sgRNA-guided Cas9 for three DSB events per chromosome (Fig. 4 and Fig. S3), the synchronous tagging of multiple genes using the corresponding multi-sgRNAs could theoretically be obtained through one-step transformation using the Cas9-based tool. When tagging more than one gene, especially the essential genes, the Cas9-based tool would be much easier and less time-consuming than the conventional methods, which would take weeks or even years and require laborious work. The easy *in situ* tagging method developed in this work will accelerate studies on specific gene expression, immunoprecipitation to determine specific protein-protein and protein-DNA/RNA interactions or networks, and purification of proteins in their native states that may contain specific or novel posttranslational modifications.

Currently, there are no applicable methods for performing *in situ* point mutagenesis in *M. maripaludis* (29, 49), thus limiting studies on the functions of key residues in genes and proteins in the *in situ* chromosomal context. In this work, using the Cas9-based tool, *in situ* nucleotide substitutions, for both multiple nucleotides and even single nucleotides, have been achieved. This will provide a foundation for understanding the key elements and sites of genes or intergenic regions through introducing multiple- or single-nucleotide polymorphisms. Indeed, using a Cas9-based strategy through one-step transformation, we easily generated multinucleotide mutations on the polyuridines of the terminator of *MMP0290* and further determined their function in transcription termination, which also led to the identification of a new upstream terminator of *MMP0290*. This finding supports the key role of the terminator U-rich sequence in transcription termination, as recently reported for *M. maripaludis* (33, 34), and also suggests that consecutive terminators encoded in *M. maripaludis* and may have a regulatory function on transcription termination. Similar phenomena were found in other two archaeal representatives (58), implying that controlled transcription termination by alternative terminators may be common in archaeal organisms.

Using the one-step Cas9-based strategy, this study also successfully generated single-nucleotide mutagenesis in the polyploid chromosomes of *M. maripaludis*. This enables us to evaluate the key elements of the promoter at the *in situ* site in *M. maripaludis*, which illustrated the importance of the BRE sequence to promoter activity *in vivo* (Fig. 7A). Single-nucleotide mutagenesis using Cas9 also facilitates the successful introduction of a stop codon into a gene, thereby suppressing its expression (Fig. 7C). This provides an alternative strategy to inactivate the expression of expected genes, which can avoid the polarity effect produced by genetic manipulation. Therefore, the multiple- and single-nucleotide mutation strategies developed in *M. maripaludis* will further increase the robustness of the Cas9-based genetic system and extend its applications in archaeal biology studies and archaeal biotechnology developments.

In summary, in this study, we have developed a highly efficient, precise, and rapid CRISPR-Cas9 genome editing toolkit in an autotrophic CO_2_-fixing methanogenic archaeon, *M. maripaludis*. The system enables single- and multiple-gene knockout and genetic modifications, including gene tagging and multiple- and single-nucleotide mutagenesis. The robust genetic toolkit explored in this work will pave the way to develop *M. maripaludis* into a workhorse in archaeal biology studies and archaeal biotechnology applications, especially in the establishment of CO_2_-based biotechnology.

## MATERIALS AND METHODS

### Strains, plasmids, and culture conditions.

All the plasmids and strains used in this study are listed in Table S1. *M. maripaludis* S0001 and its derivatives were grown in prereduced McF medium under a gas phase of N_2_/CO_2_ (80:20) at 37°C as previously described (29), and 1.5% agar was used in the solid medium. Growth was monitored by determining the optical density at 600 nm (OD_600_). Puromycin (2.5 μg/mL) was used for genetic modification selections unless indicated otherwise, and the base analog 8-azahypoxanthine at 20 μg/mL was used for a counterselection for curing the Cas9-based pMEV4 plasmid as described previously (49). E. coli DH5α or BL21(DE3)/pLysS was grown at 37°C in LB broth supplemented with ampicillin (100 μg/mL) when needed.

### Plasmid construction for Cas9-based genome editing in *M. maripaludis*.

Plasmids used or constructed in this study are listed in Table S1, and the primers utilized for plasmid engineering and transformants screening are listed in Table S2. PCR amplification was performed using the high-fidelity KOD-plus DNA polymerase (TOYOBO, Japan). DNA fragments and the amplified plasmid backbone were ligated using the stepwise Gibson assembly using the ClonExpress MultiS one-step cloning kit (Vazyme).

Conventionally, plasmids for Cas9-based genome editing were constructed by introducing the codon-optimized SpCas9 downstream of the *phmvA* promoter in pMEV4 to obtain plasmid pMEV401 and then cloning a synthetic chimeric sgRNA(s) of a 20-bp sequence targeting a gene(s) and an 80-bp scaffold sequence to facilitate SpCas9 binding downstream of the promoter of P0058 derived from *MMP0058*, like was shown for sgRNA targeting the ORF region of *Mmp1197* (*ssuC*) (Fig. 1B). The two homologous arms (U and D, for upstream and downstream, respectively) of the donor DNA were separately amplified and inserted upstream of the sgRNA cassette through the stepwise Gibson assembly. For multiplex gene editing, the sgRNAs were separately expressed under the control of indicated gene promoters and terminators that shown in related figures and Table S3. Donors of the homologous arms for each target were constructed upstream of the sgRNAs through Gibson assembly. The constructed plasmids were validated by DNA sequencing prior being transformed into *M. maripaludis* S0001.

### PEG-mediated transformation of *M. maripaludis*.

The SpCas9-based pMEV4 plasmids were transformed into *M. maripaludis* S0001 using the polyethylene glycol (PEG)-mediated transformation approach as described previously (29, 46, 59). First, transformation buffer (TB) and TB-PEG were prepared as follows. For TB, 50 mM Tris base, 0.35 M sucrose, 0.38 M NaCl, 0.00001% resazurin, and 1 mM MgCl_2_ were mixed in a 100-mL beaker. For TB-PEG, 40% (wt/vol) PEG 8000 was added. The pH was adjusted to 7.5 using HCl and the buffer was transferred to a 50-mL serum bottle. A cysteine/dithiothreitol (DTT) solution (2.5% cysteine hydrochloride, 50 mM DTT) was prepared in a small serum bottle. After adjustment of the pH to 7.5 with Tris base, the bottle was placed in an anaerobic chamber. Gases were exchanged by sparging with N_2_ for 1 and 3 h for TB and TB-PEG, respectively. The serum bottles were transferred inside the chamber, and 1 mL of 50 cysteine/DTT solution was added to 50 mL of TB or TB-PEG. The solutions were incubated unclosed overnight or until the resazurin turned colorless. TB (or TB-PEG) was filter sterilized using disposable 0.2-mm filters, and 5-mL aliquots were transferred into sterile Balch tubes. The tubes were sealed with serum stoppers and secured with aluminum seals.

### Western blot assay.

Western blot assays to detect the expression of SpCas9, aCPSF1b, and aCPSF2 were performed similarly to that described previously (33). After resuspension in lysis buffer (20 mM Tris base, 150 mM NaCl, 10% [vol/vol] glycerol, 0.1% [vol/vol] Triton X-100), cells of *M. maripaludis* were lysed by sonication. The cell debris was precipitated by centrifugation at 10,000 × *g* for 10 min at 4°C, and the supernatant was quantified for the protein and then used for the Western blot assay. Proteins in the supernatant were separated by SDS-PAGE and then transferred to nitrocellulose membranes. Western blotting was performed using the commercial polyclonal rabbit antiserum raised against Cas9 and the customized polyclonal rabbit antiserum raised against the purified recombinant aCPSF1b and aCPSF2. The antibodies were used at a 1:5,000 dilution. Immune-active bands were visualized by an Amersham ECL Prime Western blot detection reagent (GE Healthcare) on a Tanon-5200 multichemiluminescence/fluorescence imaging system. The quantification of Western blot band intensity was performed using ImageJ.

### Northern blot assay.

The Northern blot assay was performed using the procedure described previously (33). Briefly, total RNA was extracted from mid-log-phase cells and purified using TRIzol reagent (Invitrogen). After quantification using a NanoPhotometer spectrophotometer (Implen), the total RNA was denatured for 10 min at 65°C in loading buffer containing 95% (vol/vol) formamide, and 2 to 5 μg was loaded to each lane of a 6% polyacrylamide gel with 7.6 M urea. The gel was run in 0.5× Tris-borate-EDTA (TBE) buffer. A single-stranded RNA (ssRNA) ladder (New England Biolabs) served as a size marker on denaturing polyacrylamide-urea gels. After separation, RNAs were transferred onto Hybond N+ membranes (GE Healthcare) by electroblotting or capillary blotting and cross-linked to the membrane using UV. Membranes were prehybridized at 42°C, followed by hybridization with 2 to 10 pmol of biotin-labeled DNA probes listed in Table S4 for 12 h. After three rounds of washing for 10 min each in 1×, 0.2×, and 0.1× SSC–0.1% SDS solutions (1× SSC is 0.15 M NaCl plus 0.015 M sodium citrate), signals were visualized using a chemiluminescent nucleic acid detection module (Thermo Scientific) according to the manufacturer’s protocol.

### Construction of His-Flag-tagged *Mmp-aCPSF1* and His-Flag-tagged *Mmp-RpoL* strains.

Homology arms of about 1,000 bp were PCR amplified from S0001 genomic DNA and cloned into pMD19-T to construct plasmid pMD19-T-*aCPSF1*. His_6_ and 3× Flag tags were fused to the C terminus of aCPSF1 via PCR amplification to construct plasmid pMD19-T-*aCPSF1-H2F*. sgRNA targeting the C terminus of *aCPSF1* was cloned into pMEV4-*Cas9* via PCR amplification and stepwise Gibson assembly to construct plasmid pMEV4-*Cas9-sg2*. Next, DNA fragments were amplified from pMD19-T-*aCPSF1-H2F* using primers C1-HF-F/C1-HF-R and fused with pMEV4-*Cas9-sg2* via NEBuilder HiFi DNA assembly master mix to obtain plasmid pMEV4-*Cas9-sg2-aCPSF1-H2F*. Plasmid pMEV4-*Cas9-sg2-aCPSF1-H2F* was transformed into S0001 to obtain an *Mmp-aCPSF1-H2F*strain . pMEV4-*Cas9-sg3- RpoL-H2F* was constructed and the *Mmp-RpoL-H2F* strain was obtained similarly. Additionally, synonymous codon mutations were introduced into the donor of *aCPSF1* in plasmid pMEV4-*Cas9-sg2-aCPSF1-H2F* to avoid Cas9 cutting of the donor.

### Construction of the plasmids for *in situ* multiple- or single-nucleotide mutagenesis.

First, a strain *M. maripaludis* carrying an mCherry gene driven by the promoter P0386 encoded in the genome was constructed using a method described previously (34); the strain is referred to as the *Mmp-mCherry* strain (Table S1). To generate the plasmids for single-nucleotide mutagenesis at the promoter region of P0386, one sgRNA, *sgP0386*, targeting the promoter region was cloned into pMEV4-*Cas9* (pMEV401) via PCR amplification and Gibson assembly to obtain plasmid pMEV4-*Cas9-sgP0386* (Table S1). The ~1,000-bp homology arms were amplified from the genomic DNA of the *Mmp-mCherry* strain via PCR and fused upstream of the *sgP0386* sgRNA sequence with the pMEV4-*Cas9-sgP0386* backbone via Gibson assembly using NEBuilder HiFi DNA assembly master mix to obtain plasmid pMEV4-*Cas9-sgP0386-P0386-mCherry* (Table S1). Next, mutagenesis of different single nucleotides of the P0386 promoter region, which is also in the *sgP0386* sgRNA targeting region of the donor sequence, was achieved via the site-directed gene mutagenesis kit based on PCR amplification and DpnI digestion. Thus, a series of plasmids, pMEV4-*Cas9-sgP0386-P0386(-3)-mCherry*, pMEV4-*Cas9-sgP0386-P0386(-2)-mCherry*, pMEV4-*Cas9-sgP0386-P0386(-2 and -3)-mCherry*, pMEV4-*Cas9-sgP0386-P0386(1)-mCherry*, and pMEV4-*Cas9-sgP0386-P0386(3)-mCherry*, were obtained and transformed to strain S0001 to obtain the *Mmp-P0386(-3)-mCherry*, *Mmp-P0386(-2)-mCherry*, *Mmp-P0386(-2, -3)-mCherry*, *Mmp-P0386(1)-mCherry*, and *Mmp-P0386(3)-mCherry* strains (Table S1). To introduce the stop codon into the mCherry gene in the *Mmp-mCherry* strain, plasmids pMEV4-*Cas9-sg5-(-2)mCherry* and pMEV4-*Cas9-sg8-(1m)mCherry* were constructed, and the *Mmp-sg5(-2m)-mCherry* and *Mmp-sg8(1m)-mCherry* strains were obtained with a similar method. Moreover, to achieve the multiple-nucleotide mutagenesis in the polyuridines of the terminator of gene *MMP0290*, the plasmid and the related strain were constructed with a method similar to that described for the single-nucleotide mutagenesis except that multiple nucleotides to mutate the polyuridines were generated at the donor sequence.

### Fluorescence intensity assay.

Expression of the mCherry reporter gene in the *Mmp-mCherry* strain and the related mutated strains was quantified as described previously (34, 35). Briefly, colonies were cultured to exponential phase and cell density (OD_600_) was measured. Cells from 1 mL of culture were collected at 4°C by centrifugation at 17,900 × *g*. Then the cells were resuspended, lysed with 200 μL of 20 mM PIPES [piperazine-*N*,*N*′-bis(2-ethanesulfonic acid)], and then shaken for 12 h at 30°C and 220 rpm. The samples were withdrawn and centrifuged at 30°C and 17,900 × *g*. Next, the fluorescence of the supernatant with appropriate volume was measured on a Synergy H4 hybrid multimode microplate reader (BioTek Instruments) with excitation and emission wavelengths of 575 and 610 nm, respectively. The fluorescence intensity of mCherry was defined as the ratio of detected fluorescence units (RFUs) divided by the optical density (OD_600_) and the sample volume.
