# Supplementary material for: CRISPR-Cas9 Toolkit for Genome Editing in an Autotrophic CO2-Fixing Methanogenic Archaeon
Source: Microbiol Spectr. 2022 Jun 29;10(4):e01165-22. doi: 10.1128/spectrum.01165-22 (PMC9430280; doi:10.1128/spectrum.01165-22)
Supplement: Supplemental file 1 — Supplemental material. Download spectrum.01165-22-s0001.pdf, PDF file, 0.6 MB [file spectrum.01165-22-s0001.pdf]

Supplementary Information for

CRISPR-Cas9 toolkit for genome editing in an autotrophic CO_2 fixing methanogenic archaeon

This file contains:

Supplementary Figures S1-S5.

Supplementary Tables S1-S5.

Supplementary Figures

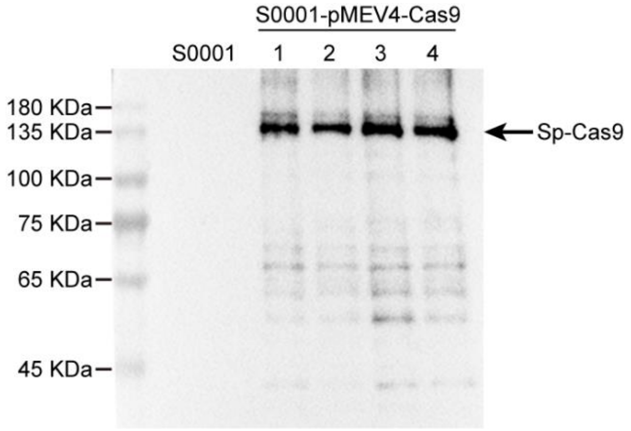

Figure S1. The Cas9 expression in *M. maripaludis*. The optimized SpCas9 was constructed in pMEV4 plasmid and transferred into *M. maripaludis* strain S0001 and its expression was verified by western blot using the commercial antibody of SpCas9. Arrow indicated the protein band of the soluble SpCas9. Lanes 1 to 4 display protein samples from four selected transformants after puromycin selection.

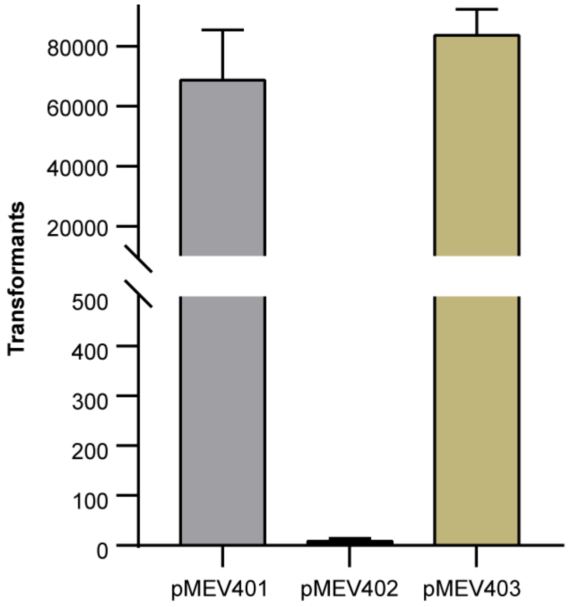

Figure S2. The numbers of puromycin resistant transformants obtained by separately transferring 1 μg plasmid DNA of pMEV401, 402, and 403 into *M. maripaludis*.

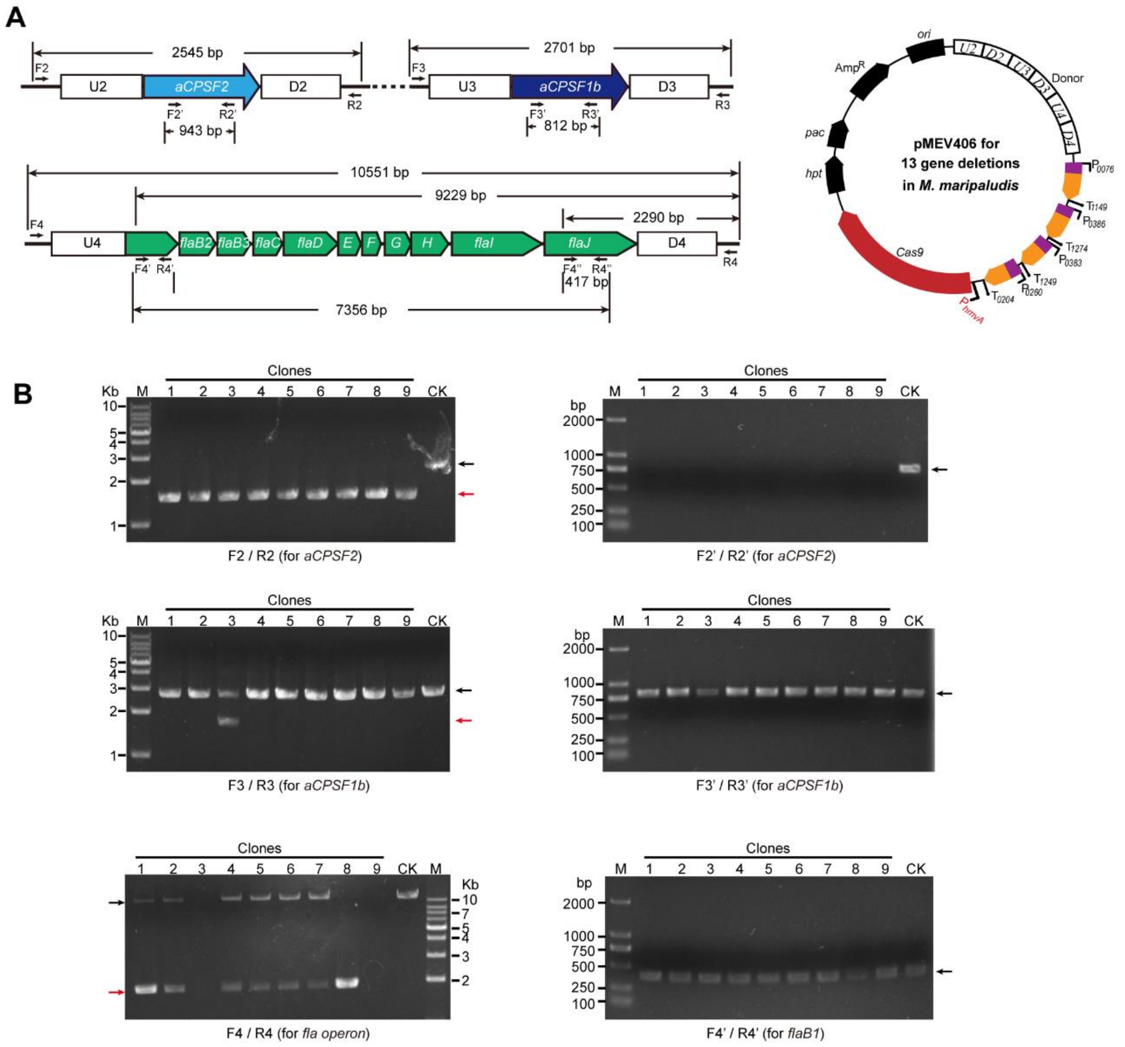

Figure S3. Synchronous deletion of multiple (13) genes located at three different genomic loci using the Cas9-editing system explored in *M. maripaludis* by one-step transformation. (A) The design of simultaneous deletion of the multiple genes including two β-CASP ribonucleases encoding genes, *aCPSF2* (*MMP0431*) and *aCPSF1b* (*MMP1381*), and the whole *fla* operon genes of *MMP1666* to *MMP1676*. The pMEV406 plasmid encodes four sgRNAs that respectively match *aCPSF2* (*MMP0431*), *aCPSF1b* (*MMP1381*), *flaB1* (*MMP1666*) and *flaJ* (*MMP1676*), which were expressed by indicated promoters derived from highly expressed genes in *M. maripaludis*. The sequences upstream (U2, U3, and U4) and downstream (D2, D3, and

D4) of each target gene were concatenated on pMEV406 to provide the recombinant donor. Distinctly, the Cas9 expressed in pMEV406 plasmid was derived by a promoter of P*hmvA*, a constitute promoter commonly used in *M. maripaludis*. (B) Ten puromycin resistance transformants were randomly selected for PCR analysis with primers F2/ R2, F2'/R2', F3/R3 F3'/R3', F4/R4, and F4'/R4' to detect the knock out of the *aCPSF2* and *aCPSF1b* genes, and the *fla* operon genes, respectively. Black and red arrows indicate the PCR products amplified from the wild-type (CK) and the mutated genomes, respectively. M, dsDNA size marker.

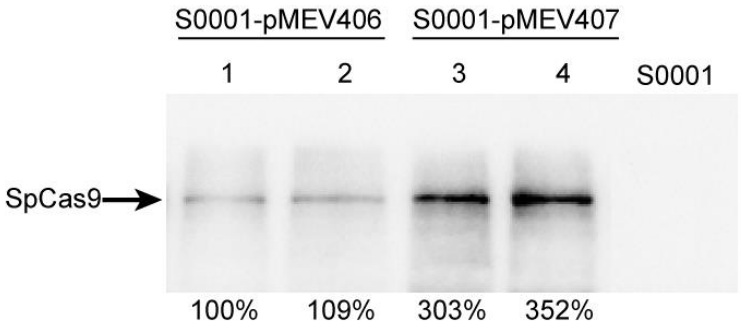

Figure S4. Comparison of SpCas9 expression in pMEV406 and pMEV407. Using the commercial antibody of SpCas9, Western blot assayed improved expression of SpCas9 under a stronger promoter P0386 in pMEV407 compared with that under PhmvA in pMEV406. Arrow indicats the soluble SpCas9 protein. By reference to the band intensity (100%) of SpCas9 expressed in pMEV401, the protein percentages expressed in strain S0001 carrying pMEV406 and pMEV407 were calculated.

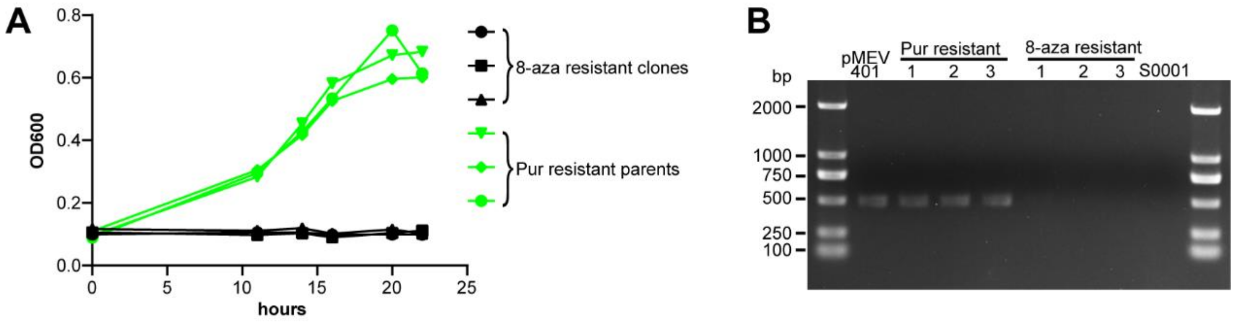

Figure S5. Curing of the Cas9-based pMEV4 plasmid using the *hpt* counterselection marker. (A) Growth curves of three puromucin-resistant (Pur^R) transformants and three 8-aza resistant transformants derived from the Pur^R parent after curing the plasmid were compared in liquid medium with 2.5 µg/ml puromycin. (B) Primers to amplify the hpt gene in the pMEV4 plasmid were used to screen for the completeness of the curing for the Cas9-based pMEV4 plasmid in each of the three Pur^R parents (lanes Pur resistant) and 8-azahypoxanthine resistant transformants (lanes 8-aza resistant). Plasmid pMEV4 and the parental strain S0001 were used as positive and negative controls, respectively (lanes pMEV401 and S0001).

Supplementary Tables

Table S1. Strains and plasmids used in this study

Strains and plasmids	Characteristics and descriptions	Reference or sources
**Strains**		
E. coli DH5α	F^{-}φ80d *lac*ZΔM15 Δ(*lac*ZYA-*arg* F) U169 *end*A1 *rec*A1 *hsd*R17(r_k^-,m_k^+) *sup*E44λ- *thi* -1 *gyr*A96 *rel*A1 *pho*A	TransGen , Beijing
M. maripaludis S0001	S2 Δhpt + ORF1 of pAW42	(1)
Mmp-ΔMMP1197	S0001 with *Mmp1197* deletion	This study
Mmp-ΔaCPSF1b/aCPSF2	S0001 with *aCPSF1b* and *aCPSF2* deletion	This study
Mmp-Δfla	S0001 with *fla* operon deletion	This study
Mmp-ΔaCPSF1b/aCPSF2/Δfla	S0001 with *aCPSF1b*、*aCPSF2 and fla* operon deletion	This study
Mmp-mCherry	S0001 with mCherry inserted into the intergenic region of *MMP0852*/*MMP0853* (*MMP0852*:: P*mcr-sat*-T*mcr, MMP0853*:: P*0386-mCherry*-$T_{mmp1149}$)	This study
Mmp-aCPSF1-H2F	S0001 with *MMP0694* 3′-end His_6 and 3Flag tags	This study
Mmp-muaCPSF1-H2F	S0001 with *MMP0694* 3′-end His_6 and 3Flag tags	This study
Mmp- RpoL-H2F	S0001 with *MMP0261* 3′-end His_6 and 3Flag tags	This study
Mmp- P0386(-3)-mCherry	Mmp-mCherry with -2 point mutagenesis of *P0386*	This study
Mmp- P0386(-2)-mCherry	Mmp-mCherry with -2 point mutagenesis of *P0386*	This study
Mmp- P0386(-2、*-3)-mCherry*	Mmp-mCherry with -2 and -3 point mutagenesis of *P0386*	This study
Mmp- P0386(1)-mCherry	Mmp-mCherry with 1 point mutagenesis of *P0386*	This study
Mmp- P0386(3)-mCherry	Mmp-mCherry with 3 point mutagenesis of *P0386*	This study
Mmp- sg5(-2m)-mCherry	Mmp-mCherry with -2 point mutagenesis of *sg5*	This study
Mmp- sg8(1m)-mCherry	Mmp-mCherry with 1 point mutagenesis of *sg8*	This study
Mmp-T0290-mutant	S0001 with multiple mutagenesis of terminator of *MMP0290* (T1149)	This study
**Plasmids**		
pMD19-T	Amp^R	Takara , Japan
pMD19-T-ORF-1197	pMD19-T with Mmp-1197 donor and its open reading frame (ORF)	This study
pMD19-T-Donor-1197	pMD19-T with Mmp-1197 donor	This study
pMD19-T-ORF-aCPSF1b	pMD19-T with aCPSF1b donor and its open reading frame (ORF)	This study
pMD19-T-Donor-aCPSF1b	pMD19-T with aCPSF1b donor	This study
pMD19-T-ORF-aCPSF2	pMD19-T with aCPSF2 donor and its open reading frame (ORF)	This study
pMD19-T-Donor-aCPSF2	pMD19-T with aCPSF2 donor	This study
pMEV4	*E. coli*/*M. maripaludis* shuttle vector, only replicates in *M. maripaludis* S0001 ,$Amp^{R,}$ Pur^R	This study
pMEV4-Cas9 (pMEV401)	pMEV4 containing the Cas9 ORF, $Amp^{R,}$ Pur^R	This study
pMEV4-sgRNA-Cas9 (pMEV402)	pMEV4 containing the Cas9 ORF and a guide sequence 5’-TGATTTATTTGATACAACAG targeting to *Mmp1197*, $Amp^{R,}$ Pur^R	This study

pMEV4-Cas9-△*Mmp1197* (pMEV403)	pMEV4 containing donor, Cas9 ORF and a guide sequence 5'-TGATTTATTTGATACAACAG targeting to *Mmp1197*, $Amp^{R,}$ Pur^{R}	This study
pMEV4-Cas9-△ *aCPSF1b/aCPSF2* (pMEV404)	pMEV4 containing donor, Cas9 ORF and guide sequence 5'-ATAATTATTGAATCTACTTA and 5'-TCCCTGTATTTGCAGTTGAT targeting to *aCPSF1b* and *aCPSF2,* $Amp^{R,}$ Pur^{R}	This study
pMEV4-Cas9-△*fla* (pMEV405)	pMEV4 containing donor, Cas9 ORF and a guide sequence 5'-CCGAAGAGGTAAAAATCTCA and 5'-GTAATGACTGGAAGCCTCCT targeting to *flaB1*and *flaJ,* $Amp^{R,}$ Pur^{R}	This study
pMEV4-Cas9-△ *aCPSF1b/aCPSF2/fla* (pMEV406 and pMEV407)	pMEV4 containing donor, Cas9 ORF and the guide sequences that pMEV4-Cas9-△*aCPSF1b/aCPSF2* and pMEV4-Cas9-△*fla* contians， $Amp^{R,}$ Pur^{R}	This study
pMD19-T- *aCPSF1*	pMD19-T with S0001 *MMP0694-MMP0693* fragment	This study
pMD19-T- *aCPSF1-H2F*	pMD19-T with S0001 *MMP0694*-His_6-3Flag-*MMP0693* fragment	This study
pMEV4- *Cas9-sg2- aCPSF1-H2F* (pMEV409)	pMEV4- *Cas9* with sg2 targeting *aCPSF1 and donor (MMP0694*-His_6-3Flag-*MMP0693)*	This study
pMD19-T- *RpoL*	pMD19-T with S0001 *MMP0261-MMP0260* fragment	This study
pMD19-T- *RpoL-H2F*	pMD19-T with S0001 *MMP0261*-His_6-3Flag-*MMP0260* fragment	This study
pMEV4- *Cas9-sg3- RpoL-H2F* (pMEV408)	pMEV4- *Cas9* with sg3 targeting *RpoL and donor (MMP0261*-His_6-3Flag-*MMP0260)*	This study
pMEV4-Cas9-mu0290-sg (pMEV410)	pMEV4- *Cas9* with sg targeting T1149 and *donor (MMP0290-MMP0291* with point mutagenesis of T1149 sequence*)*	This study
pMEV4- *Cas9-sgP0386-P0386(-3）-mcherry* (pMEV411)	pMEV4- *Cas9* with sg*P0386* targeting *P0386 and donor (P0386-mCherry with* -3 point mutagenesis of *sgP0386* targting sequence*)*	This study
pMEV4- *Cas9- sgP0386-P0386(-2）-mcherry* (pMEV412)	pMEV4- *Cas9* with sg*P0386* targeting *P0386 and donor (P0386-mCherry* with -2 point mutagenesis of *sgP0386* targting sequence*)*	This study
pMEV4- *Cas9- sgP0386-P0386(-2 and -3）-mcherry* (pMEV413)	pMEV4- *Cas9* with sg*P0386* targeting *P0386 and donor (P0386-mCherry* with -2 and -3 point mutagenesis of *sgP0386* targting sequence*)*	This study
pMEV4- *Cas9- sgP0386-P0386(1）-mcherry* (pMEV414)	pMEV4- *Cas9* with sg*P0386* targeting *P0386 and donor (P0386-mCherry* with 1 point mutagenesis of *sgP0386* targting sequence*)*	This study
pMEV4- *Cas9- sgP0386-P0386(3）-mcherry* (pMEV415)	pMEV4- *Cas9* with sg*P0386* targeting *P0386 and donor (P0386-mCherry* with 3 point mutagenesis of *sgP0386* targting sequence*)*	This study
pMEV4- *Cas9-sg1- (-2)mcherry* (pMEV416)	pMEV4- *Cas9* with sg*1* targeting *mCherry* and *donor (P0386-mCherry* with -2 point mutagenesis of *sg1* targting sequence*)*	This study
pMEV4- *Cas9-sg2-(1m)mcherry* (pMEV417)	pMEV4- *Cas9* with sg*2* targeting *mCherry* and *donor (P0386-mCherry* with 1 point mutagenesis of *sg2* targting sequence*)*	This study

Table S2. Primers used in this study.

Primer	Sequence (5′-3′)	Purpose
Mmp1197- genome-F	GTGACTTTGGCAGTACGCTT	Construction of strain △*Mmp*1197
Mmp1197- genome-R	CAGTCTGCCATATTTTTAAG	Construction of strain △*Mmp*1197
Del-*1197*-ORF-F	TCCTGGAAGTGATGCTGGAC	Construction of strain △*Mmp*1197
Del-*1197*-ORF-R	TCCTGTTCCAAGGATTCCAA	Construction of strain △*Mmp*1197
pMEV4-(1197)-F	GAGAATAGAAAGTACATTATATTGC	Construction of strain △*Mmp*1197
pMEV4-(1197)-R	ATAACTTAAAAAACCTTGGATTTTTG	Construction of strain △*Mmp*1197
Mmp1197-(pMEV4)-F	TCCAAGGTTTTTTAAGTTATGTGACTTTGGCAGTACGC	Construction of strain △*Mmp*1197
Mmp1197-(pMEV4)-R	ATAATGTACTTTCTATTCTCCAGTCTGCCATATTTTTAAGAGG	Construction of strain △*Mmp*1197
△- *Mmp1197*out-F（F1）	GCACAGCATGCAACAGCAAC	Verification of knockout- *Mmp1197*
△- *Mmp1197* out -R（R1）	CTTGAGTCTCAGGCTCTTCG	Verification of knockout- *Mmp1197*
△- *MMP1197* in-F（F1`）	AGCATGGGAAGCTCTTGCAA	Verification of knockout-*Mmp1197*
△-*MMP1197* in-R(R1`)	CCAGCATCACTTCCAGGAAG	Verification of knockout- *Mmp1197*
*aCPSF2*up-dn-(T)F：	CTTTCAAAACATCGTAATCTATTTTTGG	Deletion of *Mmp*（*aCPSF2*）
*aCPSF2*up-dn-(T)R：	AAAATAAATCGGAGAGTCCTTGAAA	Deletion of *Mmp*（*aCPSF2*）
*aCPSF2*del-(T)-F:	CGTCGCACGCAGAAGTTGTAAAAATATTTAAAGATTG	Deletion of *Mmp*（*aCPSF2*）
*aCPSF2*del-(T)-R:	TACAACTTCTGCGTGCGACGCAAAGACAC	Deletion of *Mmp*（*aCPSF2*）
aCPSF1b up-dn-(T)F：	CCAGATTTTCTTATCCATGCGAC	Deletion of *Mmp*（*aCPSF1b*）
aCPSF1b up-dn-(T)R：	CTGCTGTTTTTCCTTGCAG	Deletion of *Mmp*（*aCPSF1b*）
aCPSF1b del-(T)-F:	AGATAAACACGAGCGATTCCGGTAAATG	Deletion of *Mmp*（*aCPSF1b*）
aCPSF1b del-(T)-R:	GGAATCGCTCGTGTTTATCTCTACGCAAG	Deletion of *Mmp*（*aCPSF1b*）
Sg *aCPSF1b/aCPSF2*-(pMEV4)-F:	TAAAAATATGGCAGACTGGATCAAATGGTAAGGTATATATAGTG	Construction of strain △*Mmp*（*aCPSF1b/aCPSF2*）
flaJ dn-(pMEV4)-	ATATACCTTACCATTTGATCGCGGGTTTGGAAA	Construction of strain △*Mmp fla*

R:	TGCTTTAAATG	
aCPSF1b in-F（F2`）	TGCGGTGCAATACCTTACTT	Verification of knockout- *aCPSF1b*
aCPSF1b in-R(R2`)	CCTTCAGCTTGATATCCGGT	Verification of knockout- *aCPSF1b*
aCPSF1b out-F(F2)	TGGAAGACCCACTCAAAGAG	Verification of knockout- *aCPSF1b*
aCPSF1b out-R（R2）	AGGGTGAATTCCTTCTCCAC	Verification of knockout- *aCPSF1b*
aCPSF2 in-F(F3`)	GAAGGGCAGGATATCCCCTA	Verification of knockout- *aCPSF2*
aCPSF2 in-R(R3`)	AATTCGTCCATTTCGCCATG	Verification of knockout- *aCPSF2*
aCPSF2 out-F(F3)	GTGAACTACTGCTGGAAGGT	Verification of knockout- *aCPSF2*
aCPSF2 out-R(R3)	GACGGTAGCGTTGCAGTTCA	Verification of knockout- *aCPSF2*
flaB1-in F(F4`)	CTGGAAGCGTTGGAACATCA	Verification of knockout- *fla*
flaB1-in R(R4`)	ACCTGGAGCACCAAATTCTG	Verification of knockout- *fla*
flaJ-in-F (F4``)	CAGGCTACACTCGGTTGGTC	Verification of knockout- *fla*
flaJ-in-R (R4``)	CACCGTAGTCTTCCATCACG	Verification of knockout- *fla*
Fla-out-F (F4)	CAACGAGTGTATCAGTTGCA	Verification of knockout- *fla*
Fla-out-R (R4)	AATTCTCGCATCAATAGTCG	Verification of knockout- *fla*
P0386-newRBS-Cas9-F	TTTTAAACGATTTTTCCGAAAGATTTATATATTTAGTTTTCCAAAATTAAACTGGATAACTAATACTAGAGAGGAGGTGAAATAATGGATAAAAAATATAGTATTGGTTTAGATATAGG	Construction of strain Δ*Mmp aCPSF1b/aCPSF2/fla*
P0386-newRBS-Cas9-R	ATTTTTTATCCATTATTTCACCTCCTCTCTAGTATTAGTTATCCAGTTTTAATTTTGGAAAACTAAATATATAAATCTTTCGGAAAAATCGTTTAAAAATAAAAAAAAGTAGTATATTTATATATTTCGCACCGACTCG	Construction of strain Δ*Mmp aCPSF1b/aCPSF2/fla*
P0386-oldRBS-F	TATTTTTAAACGATTTTTCCGAAAGATTTATATATTTAGTTTTCCAAAATTAAAACTGACTAGAGTGCAGGTAGCGCTATGG	Construction of strain Δ*Mmp aCPSF1b/aCPSF2/fla*
P0386-oldRBS-R	CCTGCACTCTAGTCAGTTTTAATTTTGGAAAACTAAATATATAAATCTTTCGGAAAAATCGTTTAAAAATAAAAAAAAGTAGTATATTTATATATTTC	Construction of strain Δ*Mmp aCPSF1b/aCPSF2/fla*
H2F-F	GAGATCTCACCACCACCACCACCACGAAAATGATTATAAAGATGACGATGACAAAGATTATAAAGATGACGATGACAAAGATTATAAAGATGACGATGACAAATAAGTATTCTTTTTTTATTTTTAATTC	Construction of in situ tagging of *aCPSF1*
H2F-R	AAAGAATACTTATTTGTCATCGTCATCTTTATAATCTTTGTCATCGTCATCTTTATAATCTTTGTCATCGTCATCTTTATAATCATTTTCGTGGTGGTGGTGGTGGTGAGATCTCAATCTTATTGAATCG	Construction of in situ tagging of *aCPSF1*
Sg2-anti0694tem-(Cas9-pMEV4)-F	ATTATACTTGCTTATTGAATCGAGATTCATGTTTTAGAGCTAGAAATAGCAAG	Construction of in situ tagging of *aCPSF1*
Sg2-anti0694tem-(Cas9-pMEV4)-R	GCTCTAAAACATGAATCTCGATTCAATAAGCAAGTATAATTACTAATCAGCAATATAATG	Construction of in situ tagging of *aCPSF1*
C1-HF-F	TCCAAGGTTTTTTAAGTTATCCTCGCAGATGATGAGAAAAAC	Construction of in situ tagging of *aCPSF1*
C1-HF-R	ATAATGTACTTTCTATTCTCCCTGCTTCTTCTAAT	Construction of in situ tagging of *aCPSF1*

	TGGG	
pMEV-sg-Cas9-F	GAGAATAGAAAGTACATTATATTGC	Construction of in situ tagging of *aCPSF1*
pMEV-sg-Cas9-R	ATAACTTAAAAAACCTTGGATTTTTG	Construction of in situ tagging of *aCPSF1*
mutant-0694tem -F	CGAAAGCTCCAATGAATTTAGATTCAATAAGATTGAGATCTCACCAC	Construction of in situ tagging of *aCPSF1*
mutant-0694tem -R	TGAGATCTCAATCTTATTGAATCTAAATTCATTGGAGCTTTCGTTTC	Construction of in situ tagging of *aCPSF1*
yz-C1-F（F6）	GGACACATTTTAGGTTCAGC	Validation of in situ tagging of *aCPSF1*
yz-0693-R（R6）	CCTTTCAAGAACTAGTTCTCCCG	Validation of in situ tagging of *aCPSF1*
RpoLC-（S0001）-F	ATTCTTGACATTTATTCTGAAC	Construction of in situ tagging of *RpoL*
RpoLC-（S0001）-R	CGATAAAGAAGCCGTGTG	Construction of in situ tagging of *RpoL*
H2F-RpoLC-F	TTTACACCACCACCACCACCACGAAAATGATTATAAAGATGACGATGACAAAGATTATAAAGATGACGATGACAAAGATTATAAAGATGACGATGACAAATAATTGATATTTTATATCGGCATAATTTAT	Construction of in situ tagging of *RpoL*
H2F-RpoLC-R	TATCAATTATTTGTCATCGTCATCTTTATAATCTTTGTCATCGTCATCTTTATAATCTTTGTCATCGTCATCTTTATAATCATTTTCGTGGTGGTGGTGGTGGTGTAAATCTTCAAGGGTTTTGTTAC	Construction of in situ tagging of *RpoL*
SgRpoLCtem-3-(Cas9-pMEV4)-F	ATTATACTTGTATCAATTATAAATCTTCAAGTTTTAGAGCTAGAAATAGCAAG	Construction of in situ tagging of *RpoL*
SgRpoLCtem-3-(Cas9-pMEV4)-R	GCTCTAAAACTTGAAGATTTATAATTGATACAAGTATAATTACTAATCAGCAATATAAT	Construction of in situ tagging of *RpoL*
RpoLC-(pMEV4-Cas9)-F	TCCAAGGTTTTTTAAGTTATATTCTTGACATTTATTCTGAACTCAAC	Construction of in situ tagging of *RpoL*
RpoLC-(pMEV4-Cas9)-R	ATAATGTACTTTCTATTCTCCGATAAAGAAGCCGTGTGCG	Construction of in situ tagging of *RpoL*
yz-RpoL-F（F5）	GGCGATGAAAAGTACAAACTCGTAGAAGACC	Validation of in situ tagging of *RpoL*
yz-2060-R（R5）	GTTTGCATTTGCAGGCACTGGTTGTGGC	Validation of in situ tagging of *RpoL*
Sg0386tem-(Cas9-pMEV4)-F	ATTATACTTGAACTAAATATATAAATCTTTGTTTTAGAGCTAGAAATAGCAAG	Construction of in situ point mutagenesis of *P0386*
Sg0386tem-(Cas9-pMEV4)-R	GCTCTAAAACAAAGATTTATATATTTAGTTCAAGTATAATTACTAATCAGCAATATAAT	Construction of in situ point mutagenesis of *P0386*
Sat-P0386-mCherry-*(Cas9-pMEV4)*-F	TCCAAGGTTTTTTAAGTTATCTTTAGTTAATGGTGGATCAACTGG	Construction of in situ point mutagenesis of *P0386*
Sat-P0386-mCherry-*(Cas9-pMEV4)*-R	ATAATGTACTTTCTATTCTC TGATGCTTCCCAACCCATTG	Construction of in situ point mutagenesis of *P0386*
P0386-negative3-F	TTTATTAACCTATTTTTTTTTTCGAAAGATTTATATATTTAG	Construction of in situ point mutagenesis of *P0386*
P0386-negative3-R	CTAAATATATAAATCTTTCGAAAAAAAAAATAGGTTAATAAA	Construction of in situ point mutagenesis of *P0386*
P0386-negative2-F	TTTATTAACCTATTTTTTTTT CTGAAAGATTTATATATTTAG	Construction of in situ point mutagenesis of *P0386*
P0386-negative2-R	CTAAATATATAAATCTTTCAGAAAAAAAAAATAGGTTAATAAA	Construction of in situ point mutagenesis of *P0386*
P0386-negative 2、3-F	TTTATTAACCTATTTTTTTTT TTGAAAGATTTATATATTTAG	Construction of in situ point mutagenesis of *P0386*
P0386-negative 2、3-R	CTAAATATATAAATCTTTCAAAAAAAAAAAATAGGTTAATAAA	Construction of in situ point mutagenesis of *P0386*
P0386-1-F	TAACCTATTTTTTTTTCCGGAAGATTTATATATTTAGTTTTCC	Construction of in situ point mutagenesis of *P0386*
P0386-1-R	GGAAAACTAAATATATAAATCTTCCGGAAAAAAAAATAGGTTA	Construction of in situ point mutagenesis of *P0386*
P0386-3-F	CCTATTTTTTTTTCCGAAGGATTTATATATTTAGTTTTCC	Construction of in situ point mutagenesis of *P0386*
P0386-3-R	GGAAAACTAAATATATAAATCCTTCGGAAAAAA	Construction of in situ point mutagenesis of *P0*

	AAATAGG	*386*
SAT-F	GTGTCCTGCTCCTCTTTCTCTAGC	Validation of in situ point mutagenesis of *P0386*
MCH-R	AACGTTGTATGCACCTGGTAACTGAACTG	Validation of in situ point mutagenesis of *P0386*
Sg5-mCherry -F	ATTATACTTGGTGGTCCATTACCATTCGCAGTTTTAGAGCTAGAAATAGCAAG	Construction of in situ point mutagenesis of *mCherry*
Sg5-mCherry -R	GCTCTAAAACTGCGAATGGTAATGGACCAC CAAGTATAATTACTAATCAGCAATATAAT	Construction of in situ point mutagenesis of *mCherry*
temsg8-mCherry -F	ATTATACTTG TTTGAACCGTACATAAACTGGTTTTAGAGCTAGAAATAGCAAG	Construction of in situ point mutagenesis of *mCherry*
temsg8-mCherry -R	GCTCTAAAACCAGTTTATGTACGGTTCAAA CAAGTATAATTACTAATCAGCAATATAAT	Construction of in situ point mutagenesis of *mCherry*
mCherry sg1-pam （-2） TAG -F2	CCATTCGCATAGGATATATTATCACCACAGTTTATGTACGGTTC	Construction of in situ point mutagenesis of *mCherry*
mCherry sg1-pam （-2） TAG -R2	CTGTGGTGATAATATATCCTATGCGAATGGTAATGGACCACC	Construction of in situ point mutagenesis of *mCherry*
mCherry temsg2-1TAG -F2	GCATGGGATATATTATCACCATAGTTTATGTACGGTTCAAAAGCATACG	Construction of in situ point mutagenesis of *mCherry*
mCherry temsg2-1TAG -R2	GCTTTTGAACCGTACATAAACTATGGTGATAATATATCCCATGCG	Construction of in situ point mutagenesis of *mCherry*
Mmp0290- genome-F	TTGGAATCCTTGGAACAGGACGAAGATTTAAAGGCTGTC	Construction of strain *Mmp-T0290-mutant*
Mmp0291- genome-R	ATAATGTACTTTCTATTCTCATGGAAAATCACTTAATCTTGAG	Construction of strain *Mmp-T0290-mutant*
mutant-T*1149* -F	TTTTTTAATTAAAGAATACACTACATACACCAATAAGAAATACTCATCTAAGGCCGGTGAGAGAATG	Construction of strain *Mmp-T0290-mutant*
mutant-T*1149* -R	CGGCCTTAGATGAGTATTTCTTATTGGTGTATGTAGTGTATTCTTTAATTAAAAAAATTAAAAATTAGTTTCCGAG	Construction of strain *Mmp-T0290-mutant*
Sg-T1149-F	ATTATACTTGGTGAGAGAATGGATATCGAAGTTTTAGAGCTAGAAATAGCAAG	Construction of strain *Mmp-T0290-mutant*
Sg-T1149-R	GCTCTAAAACTTCGATATCCATTCTCTCACCAAGTATAATTACTAATCAGCAATATAATG	Construction of strain *Mmp-T0290-mutant*

Table S3. Sequences of promoters and terminators used to express sgRNAs in this study.

Plasmid	sgRNA	Promoters(5`-3`)	Terminators(5`-3`)
pMEV402<br>pMEV403	*ssuC(1197)*	Pmcr:<br>GAATAGAAAGTACATTATATTGCTGATTAGTAATTATACTTG	T1149:<br>TTATTTTTTTACTTTTTTTAAATTTTAAA
pMEV404	*aCPSF1b(1381)*	P0383:<br>TCAAATGGTAAGGTATATATAGTGATGGCGACAAGGCTATTCTTG	T1249:<br>AAAACTTAATCTAATACTTTTTTTAATTTTTGAAAT
	aCPSF2(0431)	P1555:<br>TTTAATGAAAACTTGAATATATATTCTTTCAGTAATGTTATGATG	T1274:<br>TTAAAAGAATAATTCTTTTTTTTATTTTTTTAACTG
pMEV405	*flaB1(1666)*	P0383<br>TCAAATGGTAAGGTATATATAGTGATGGCGACAAGGCTATTCTTG	T1249:<br>AAAACTTAATCTAATACTTTTTTTAATTTTTGAAAT
	flaJ(1676)	P1555:<br>TTTAATGAAAACTTGAATATATATTCTTTCAGTAATGTTATGATG	T1274:<br>TTAAAAGAATAATTCTTTTTTTTATTTTTTTAACTG
pMEV406<br>pMEV407	*flaB1(1666)*	P0076:<br>ATGAACTCAGATACTTATATATACTAGCAAATGAAGTGTAATATA	T1149：<br>TTATTTTTTTACTTTTTTTAAATTTTAAA
	flaJ(1676)	P0386:<br>TTTTTCCGAAAGATTTATATATTTAGTTTTCCAAAATTAAAACTG	T1274:<br>TTAAAAGAATAATTCTTTTTTTTATTTTTTTAACTG
	aCPSF1b(1381)	P0383:<br>TCAAATGGTAAGGTATATATAGTGATGGCGACAAGGCTATTCTTG	T1249:<br>AAAACTTAATCTAATACTTTTTTTAATTTTTGAAAT
	aCPSF2(0431)	P0260：<br>TTTAACTCAGTAACTTATATATAATAGTAAGTGATAGTATGTCTG	T0204:<br>TATATAAATATACTACTTTTTTTTATTTTTAAACGA
pMEV408	*RpoL(0261)*	Pmcr:<br>GAATAGAAAGTACATTATATTGCTGATTAGTAATTATACTTG	T1149:<br>TTATTTTTTTACTTTTTTTAAATTTTAAA
pMEV409	*aCPSF1(0694)*	Pmcr:<br>GAATAGAAAGTACATTATATTGCTGATTAGTAATTATACTTG	T1149:<br>TTATTTTTTTACTTTTTTTAAATTTTAAA
pMEV410	*MMP0290 terminator(T1149)*	Pmcr:<br>GAATAGAAAGTACATTATATTGCTGATTAGTAATTATACTTG	T1149:<br>TTATTTTTTTACTTTTTTTAAATTTTAAA

pMEV411~<br>pMEV417	*mCherry*	Pmcr:<br>GAATAGAAAGTACATTATATTGCTGATTAGTAATTATACTTG	T1149:<br>TTATTTTTTTACTTTTTTTAAATTTTAAA

Table S4. Probes used in northern blot assy.

MMP0290-mid-probe	GCTTTTCCAGTAATCGAATAAGTTTTATTTCCAAGCATGTCCATTACCTGAACTTTTGG
MMP0291-5'-probe	CCACTGCTTCGACAAATCTTCTTGCCCATCCTTCATCAGTGTCTTTAACTTCAATCACT

Table S5. Summary of the genome editing efficiency and precision achieved in this study.

Target	Plasmid	Selected transformants	Positive transformants	Editing efficiency* (Precision)
suuC	pMEV403	18	18	100%
aCPSF1b、*aCPSF2*	pMEV404	16	12	75%
fla	pMEV405	10	10	100%
aCPSF1b、*aCPSF2*、*fla*	pMEV407	9	9	100%
RpoL	pMEV408	5	5	100%
aCPSF1	pMEV409			100%
T1149	pMEV410	5	5	100%
-3 point mutagenesis of *P0386*	pMEV411	5	4	80%
-2 point mutagenesis of *P0386*	pMEV412	5	5	100%
-3 and -2 point mutagenesis of *P0386*	pMEV413	5	5	100%
1 point mutagenesis of *P0386*	pMEV414	5	5	100%
3 point mutagenesis of *P0386*	pMEV415	5	3	60%
mCherry	pMEV416~ pMEV417	5	5	100%

* The editing efficiency was calculated as the positive transformant numbers divided by the selected transformant numbers, and the editing precision was determined by the accuracy of the positive transformants by PCR product sequencing.

Reference

1. Lyu Z, Shao N, Chou CW, Shi H, Patel R, Duin EC, Whitman WB. 2020. Posttranslational methylation of arginine in methyl coenzyme M reductase has a profound impact on both Methanogenesis and growth of *Methanococcus maripaludis*. J Bacteriol 202: e00654-19.
